# Europe-wide precipitation projections at convection permitting scale with the Unified Model

**DOI:** 10.1007/s00382-020-05192-8

**Published:** 2020-06-25

**Authors:** Steven C. Chan, Elizabeth J. Kendon, Ségolène Berthou, Giorgia Fosser, Elizabeth Lewis, Hayley J. Fowler

**Affiliations:** 1 School of Engineering, Newcastle University, Newcastle upon Tyne, UK; 2 Met Office Hadley Centre, Exeter, UK

## Abstract

For the first time, we analyze 2.2 km UK Met Office Unified Model convection-permitting model (CPM) projections for a pan-European domain. These new simulations represent a major increase in domain size, allowing us to examine the benefits of CPMs across a range of European climates. We find a change to the seasonality of extreme precipitation with warming. In particular, there is a relatively muted response for summer, which contrasts with much larger increases in autumn and winter. This flattens the hourly extreme precipitation seasonal cycle across Northern Europe which has a summer peak in the present climate. Over the Western Mediterranean, where autumn is the main extreme precipitation season, there is a regional increase in hourly extreme precipitation frequency, but local changes for lower precipitation thresholds are often insignificant. For mean precipitation, decreases are projected across Europe in summer, smaller decreases in autumn, and increases in winter; comparable changes are seen in the driving general circulation model (GCM) simulations. The winter mean increase is accompanied by a large decrease of winter mean snowfall. Comparing the driving GCM projections with the CPM ones, the CPMs show a robust enhanced intensification of precipitation extremes at the convection-permitting scale compared to coarser resolution climate model projections across various European regions for summer and autumn.

**Electronic supplementary material:**

The online version of this article (10.1007/s00382-020-05192-8) contains supplementary material, which is available to authorized users.

## Introduction

The UK Met Office (UKMO) has recently completed 2.2 km continental-scale convection-permitting climate model (CPM) simulations across Europe (Berthou et al. 2018) as one of the successors to the 1.5-km CPM simulations for the Southern (SUK) or Northern UK (NUK) (Chan et al. 2018; Kendon et al. 2014). The simulation domain sizes of these older UK simulations were small, at the sub-synoptic scale—around $500 \times 500\,{\text {km}}^{2}$. The newer European-scale simulations are of slightly lower resolution compared to the original 1.5 km UK simulations, but are much more expensive computationally due to their much larger simulation domain.

Previous UK studies have shown that projections of changes to summer extreme precipitation from CPM simulations can differ substantially from those from coarser-resolution RCM simulations, but winter projections are similar (Chan et al. 2014b, 2018; Kendon et al. 2014). A similar regional-scale study also found increases in future summer precipitation extremes for Switzerland from CPM simulations (Ban et al. 2015). These summer increases have been linked to fundamental differences in how the lower-resolution parametrised-convection models and CPMs represent convective processes (Kendon et al. 2012). The CPM simulations improve the representation of the diurnal cycle (Kendon et al. 2012); lower-resolution parametrized-convection models show a dominant mid-day peak coincident with the maximum in solar shortwave heating, and do not organize convection which is important for allowing it to persist later in the day. CPMs have been shown to improve the probability distribution of hourly precipitation for some (i.e. UK and Switzerland) but not all (i.e. Germany) European regions (Berthou et al. 2018). The 1.5 km CPM simulations have fewer unrealistic “grid-point storms” that distort the representation of extreme precipitation events in the intermediate 12 km simulation (Chan et al. 2014b). The convection parametrisation scheme is designed to represent small sub-grid convection and the scheme assumptions breakdown for strong extreme-precipitation-producing grid-point-sized convective storms. Such storms at the grid-scale occur for models with grey-zone (5–20 km) resolutions but not for lower-resolution global climate and NWP models (Molinari and Dudek 1992).

The expansion of the simulation domain in this study is motivated by both operational and research experience. Operational numerical weather prediction (NWP) has shown that small domain size simulations often lead to inferior simulations due to a large spin-up region with negative precipitation biases extending considerably into the simulation domain (Lock et al., personal communication). We note that the use of an intermediate nest simulation improves the spin-up of small-scale atmospheric features, and alleviates the precipitation biases for smaller domain simulations (Fosser et al. 2019). Beyond Europe, large-domain CPM simulation has also been conducted for North America (Prein et al. 2017b) although this is a pseudo-global warming simulation (Schär et al. 1996); it is driven by reanalysis with a climate change signal superposed.

The reliability of CPM projections, along with those from all regional models, is conditional on the quality of the large-scale conditions from the driving model. For instance, much of the UK summer 1.5-km model projections are heavily conditioned by the “large” ($\approx 50\%$) reduction of summer mean precipitation from the driving general circulation model (GCM) (Chan et al. 2014b, 2018). The underlying assumption of one-way-nested downscaling is that the downscaling model does not diverge from the driving data at the synoptic scale; the added value from downscaling is found in finer spatial and temporal scales (Laprise et al. 2008). The 2.2 km CPM when driven by ERA-Interim reanalysis data (Dee et al. 2011) is able to replicate some (well-)observed European weather events (Berthou et al. 2018), demonstrating the control imposed by the driving data. One of the important new aspects of the new 2.2 km simulations are that they are driven by new and higher resolution 25-km “N512” HadGEM3 GCM simulations, which have an improved representation of the Northern Hemisphere storm track (Schiemann et al. 2017), which is in contrast with the 60-km “N216” GCM simulations that we used to drive the previous 1.5-km simulations (Mizielinski et al. 2014). Hence, we expect differences in projections between the older and newer CPM simulations simply due to the driving GCM changes. Part of our objective here is to disentangle the differences in CPM projections due to differences in the CPM itself from those due to the driving GCM, and to identify whether there is a robust signal of CPM added-value.

Here we build on Berthou et al. (2018) by examining the 2.2 km CPM climate change simulations for the first time. Readers who are interested in the Europe-wide 2.2-km ERA-Interim-driven (Dee et al. 2011) hindcast are encouraged to examine the companion publication (Berthou et al. 2018). The paper examines various improvements and limitations in the Europe-wide hindcast simulations conducted by the UKMO and ETH ${\ddot{\mathrm{Z}}}$urich; for example, improvements in diurnal cycle and Mediterranean extreme events and reduced biases in hourly precipitation intensities.

We provide a summary of the model simulations and our statistical methodologies in Sects. 2 and 3. The results are organized into three main parts: we first examine the mean precipitation biases (Sect. 4.1) and projections (Sect. 4.2) across Europe, then the changes in the frequency of occurrence of heavy precipitation events (Sect. 4.3), then the seasonal timing of such heavy precipitation events (Sect. 4.5), and lastly differences between the new results and the previous 1.5 km UK simulations (Sect. 4.6). We then draw some conclusions in Sect. 5.

## Data

**Fig. 1:**
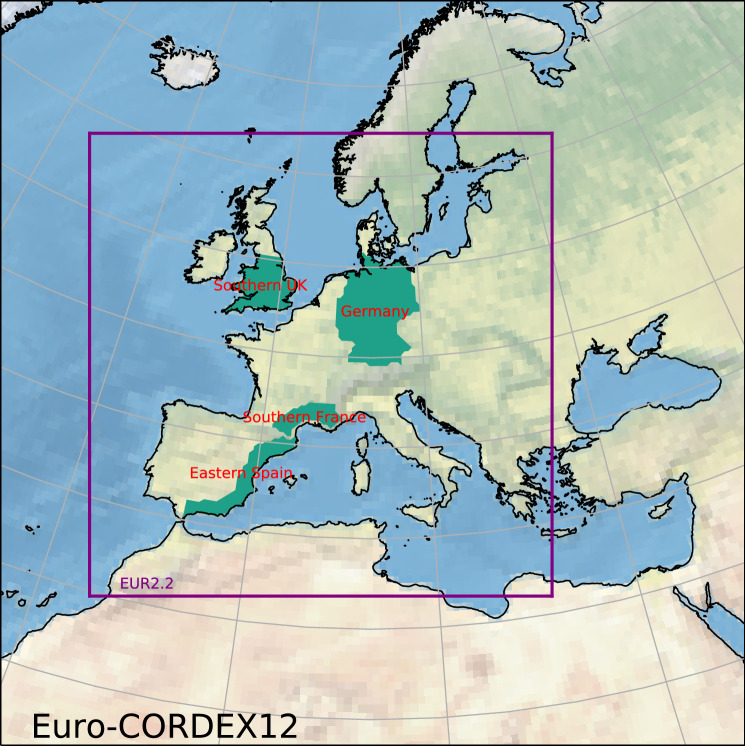
The 2.2 km CPM simulation domain within the larger Euro-CORDEX 12 km regional climate model simulation domain (Jacob et al. 2014). Our regions of interest—Germany, Southern France, Eastern Spain, and Southern UK—are labelled within

The model simulations are listed in Table 1. Results here focus on a series of 2.2 km European (Berthou et al. 2018) CPM simulations that are driven by the ERA-Interim reanalysis (“hindcast”; Dee et al. 2011) and 25-km HadGEM3 climate change simulations (Mizielinski et al. 2014). The 2.2-km CPM simulation domain is shown in Fig. 1 within the larger standard Euro-CORDEX regional climate model domain (Jacob et al. 2014).

**Table 1:** List of UKMO 2.2 km CPM and 25 km “N512” HadGEM3 GCM climate simulations that are analyzed here

Simulation	UM ver.	Domain	H-Res (km)	LBC (RCM)/SST (GCM)
2.2 km hindcast (1999–2008)	10.1	Europe	2.2	80 km ERA-Interim (Dee et al. 2011)
2.2 km present (1999–2008)	10.1	Europe	2.2	25 km HadGEM3 present (Mizielinski et al. 2014)
2.2 km future (2099–2108)${}^{\text {a}}$	10.1	Europe	2.2	25 km HadGEM3 future (Mizielinski et al. 2014)
25 km present (1999–2008)	10.3	Global	25	Daily OSTIA SST (Donlon et al. 2012)
25 km future (2099–2108)${}^{\text {a}}$	10.3	Global	25	Daily OSTIA SST + change${}^{\text {a}}$

Their UM versions, simulation domains, horizontal size of grid boxes (H-Res) are also given. The last column either gives the lateral boundary conditions (LBC) for the CPM simulations or the driving SSTs for GCM simulations. Note the hindcast simulation use the Gregorian calendar, but the present- and future-climate simulations use a 360-day calender. Monthly and seasonal means of the 360-day calender are equivalent to their Gregorian calender counterpart (Shepherd et al. 2018)

${}^{\text {a}}$RCP8.5 greenhouse gas for 2099–2108, SST 1999–2008 with monthly projected changes between 1990–2010 and 2090–2110 superposed (Mizielinski et al. 2014)

Full details of the 2.2 km simulations can be found in Berthou et al. (2018), but below we provide a few key summary points:

- The 2.2 km Europe simulations are driven by ERA-Interim and 25 km “N512” HadGEM3 present- and future-climate GCM simulations. The ERA-Interim-driven simulation has been previously examined (Berthou et al. 2018). Hence the focus here will be mostly on the GCM-driven simulations. No intermediate nest is used; the 25 km “N512” HadGEM3 and 79 km ERA-Interim data directly drives the 2.2 km CPM model with downscaling ratios of $\approx 1:11$ and 1:36, respectively.
- Recent UKMO CPM work suggests that nested simulation configurations with reduced downscaling ratios improve the ERA-Interim hindcast simulation by improving the spin up of small scale features (Fosser et al. 2019). That recommendation comes in hindsight, and is untested for different resolution jumps and for a continental-scale CPM simulation where the buffer between the lateral boundaries and area of interest (i.e. European continent) is much larger.
- The 2.2 km CPM is part of the Met Office Unified Model (UM) with model physics and configuration based on the Met Office operational UKV model (Roberts and Lean 2008) from UM version 10.1. Key features include the use of a new semi-Lagrangian dynamical core (“ENDGame”—Even Newer Dynamics for General Atmospheric Modeling of the Environment; Wood et al. 2014), and new planetary boundary layer and cloud microphysics parameterizations (Boutle et al. 2014a, b). The operational CPM also has additional near-surface stochastic potential temperature perturbations to increase convection behavior over sea. These physics changes are likely to have direct impacts on the simulated precipitation, and changes to the boundary layer and cloud microphysics parameterizations would directly change the formation and evolution of model clouds. For instance, cloud-to-rain drop autoconversion rates were too high for older model generations (including UM7.6-8) (Boutle et al. 2014a), which are hence too fast in generating rain. A scientific summary of the UM10.1 model physics can be found in Berthou et al. (2018).
- The 10-year 25 km GCM simulations include both present- (1999–2008) and future-climate (end-of-c.21) simulations. The 25 km GCM simulations use the RCP8.5 “business-as-usual” greenhouse gas scenario (RCP—Representative Concentration Pathway; Meinshausen et al. 2011). Future SSTs are observed 1999–2008 SST plus 20-year mean “delta changes” that are derived from coupled GCM simulations for 1990–2010 and 2090–2110; see Mizielinski et al. (2014) for details.
- Only common model years are examined, and we exclude the first model year to minimize the impacts from model “spin-up”. The 2.2 km hindcast simulation only covers the years from 1999 to 2008 (plus 1998 for spin up). The same applies to the climate change simulations to ensure the same underlying SSTs less the “delta changes” are used.

A number of observations are used here. For present-climate mean precipitation biases, we have used the Europe-wide E-OBS daily land-only gridded precipitation analysis between 1999 and 2008 (Cornes et al. 2018; Haylock et al. 2008). For the seasonal cycle analysis, we have used hourly non-gridded station gauge precipitation observations (GSDR; Blenkinsop et al. 2018; Lewis et al. 2019). GSDR data for Europe are only available for a few selected nations (France, Germany, Ireland, Italy (Sicily, Trentino-Alto Adige/Südtirol, and Veneto), the Low Countries (except Luxembourg), Norway, Portugal, Spain (Catalonia only), Sweden, and the UK. The data period varies, but some stations have data prior to before 1950.^1^ Depending on the analysis, gridded data are either regridded to a common 12 km (”Euro-CORDEX”) or 25 km (“N512” GCM) grid. This information will be stated specifically per analysis.

## Methods

As in Kendon et al. (2014), we have used year-block bootstrapping to estimate the significance and confidence intervals for our results. In addition, we have applied multiple-hypothesis testing, have taken the peaks-over-threshold approach in diagnosing heavy precipitation events, and have tested the statistical significance of seasonality change. A outline of the above is given in this section.

### Year-block bootstrap estimates to the* p* value and confidence intervals

Given this study uses a single model realization with a relatively small number of model years, we have only limited sampling of inter-annual variability. However, if we assume these model years are representative of the broader unknown distribution of model years, we can simulate the inter-annual variability by bootstrapping resampling (Efron and Tibshirani 1993). This approach is used in the present analysis to estimate the* p* values and confidence intervals.

To account for the temporal correlation at sub-seasonal timescales, while preserving the common underlying SST inter-annual variability, the resampling with replacement is performed in year blocks for both present- and future-climate simulations using the same resampled year indices. All bootstraps are conducted $n = 1000$ times. After bootstrapping, the* p* values are estimated in the following manner:

1. We compute the metric of our interest (i.e. the test statistic) *n* times from the bootstrap, and sort them.
2. The mean of the bootstrapped metric is computed, and is subtracted from each of *n* sorted bootstrap estimates; this creates a *n* number of 0-mean metric. This gives us an estimate of the probability distribution of the test statistic under the null hypothesis.
3. The original metric is then compared with the null distribution, and the* p* value is estimated based on where the original metric stands relative to the null distribution. For instance, if the original metric is below $2\%$ or above $98\%$ of the values in simulated null distribution, the* p* value would be $\approx 0.02$.

Note:

- Year-block resampling cannot simulate decadal or longer climate variability modes, and any meaningful analysis of such variability would require a much longer climate simulation as a prerequisite; hence, our bootstrap-estimated variability is likely to be an underestimate.
- The above assumes the probability distribution of the metric to be independent of its mean and can be translationally moved to obtain the null distribution of the test statistic.
- The smallest obtainable* p* value is $\frac{1}{n} = 0.1\%$ when the actual test statistic lies outside the bootstrapped null distribution range; $0.1\%$ is usually sufficient for statistical significance testing in the atmospheric sciences where tests are usually done at the 1–10% level.
- The 95% confidence interval of the metric is the 2.5% and 97.5% percentiles of the bootstrap-simulated metric. Such confidence intervals can be found in Fig. 4.
- The confidence intervals do not indicate statistical significance; instead, significance is indicated by the value of metric relative to the null distribution.

### Multiple hypothesis testing for spatial significance

When one conducts field significance tests, one would expect some results to be significant just by chance (Livezey and Chen 1983; Wilks 2016). The problem is further complicated by the natural spatial correlation of geophysical data, which can lead to the incorrect identification of significant results. To address this, Wilks (2016) applied corrections to the* p* value by controlling the false discovery rates in multi-hypothesis testing (Benjamini and Hochberg 1995), and then compared the corrected* p* values with a revised global significance level which is $2 \times$ the level for non-field significance tests. As the tests are two-tailed, the non-field significance level has to be halved, which cancels the $2 \times$ global significance multiplier in Wilks (2016).

We have applied the above corrections to a number of Europe-wide results: mean precipitation biases and changes, seasonality changes, and (local) precipitation extreme frequency changes. The uncorrected* p* values are estimated either parametrically or by bootstrap resampling.

These* p* value corrections are implemented in various open-source numerical analysis software: *p.adjust* in the R package *stats* (R Core Team 2016) and *stats.multitest* in the Python package *statsmodels* (Seabold and Perktold 2010).

### Peaks-over-threshold approach in diagnosing significant precipitation events

Following the peaks-over-threshold framework (Coles 2001), we define heavy precipitation events per model grid point in the present climate to be the 6-largest daily accumulated and daily 1-h maximum precipitation for all model days per model year. Therefore, for *n* model years, we select the 6*n* largest events (Blenkinsop et al. 2017). The seasonal timing information are also retained, so we can examine the change in the seasonality of these 6*n* largest events. The event “threshold” at each grid point is the smallest of the 6*n* values. We then estimate the frequency with which this event threshold is exceeded in the future-climate simulation.

The framework assumes exceedance events as part of a Poisson point process (Coles 2001). Due to the equal number of model years (to ensure the underlying SSTs are the same) and the variance of the Poisson distribution being equal to the event probability (i.e. $\sigma ^{2} = n/N = \lambda$ for *n* exceedances in *N* observations), we can then use the Z test statistic (Thode 1997) for significance testing:

1 $$\begin{aligned} Z = \frac{\lambda _{future} - \lambda _{present}}{S} = \frac{n_{future} - n_{present}}{\sqrt{n_{future} + n_{present}}} \end{aligned}$$

The uncorrected p value can then easily be obtained using the cumulative distribution function of the normal distribution, and then be adjusted for multiple hypothesis testing (Sect. 3.2).

### Changes in seasonality of exceedance events

Seasonality analysis uses the standard $\chi ^{2}$ test. For the existence of any seasonality, a one-way test is applied with the null hypothesis of no seasonality. For $N_{season}$ events for a particular season (MAM, JJA, SON, and DJF), annual frequency of $n_{\mathrm{Peaks Per Year}}$ and *T* years of data:

2 $$\begin{aligned} \chi ^{2} = \sum \limits _{\text {MAM,JJA,SON,DJF}} \frac{ (N_{season} - 0.25 n_{\mathrm{Peaks Per Year}}T)^{2} }{ 0.25 n_{\mathrm{Peaks Per Year}}T } \end{aligned}$$

For future changes, the null hypothesis is that the future seasonality is no different from the mean of both the present and future simulations. Hence, for an equal number of present- and future-climate model years:

3 $$\begin{aligned} \chi ^{2}= & {} \sum \limits _{\text {MAM,JJA,SON,DJF}} \frac{ (N_{season} - N_{season\;mean})^{2}}{ N_{season\;mean} } \end{aligned}$$

4 $$\begin{aligned} n_{season\;mean}= & {} \frac{N_{season,\;future}+N_{season,\;present}}{2}\Big |_{\mathrm{MAM,JJA,SON,DJF}} \end{aligned}$$

As the $\chi ^{2}$ test is applied to a spatial map, the multiple-hypothesis* p* value correction (Sect. 3.2) is applied.

## Results

**Fig. 2:**
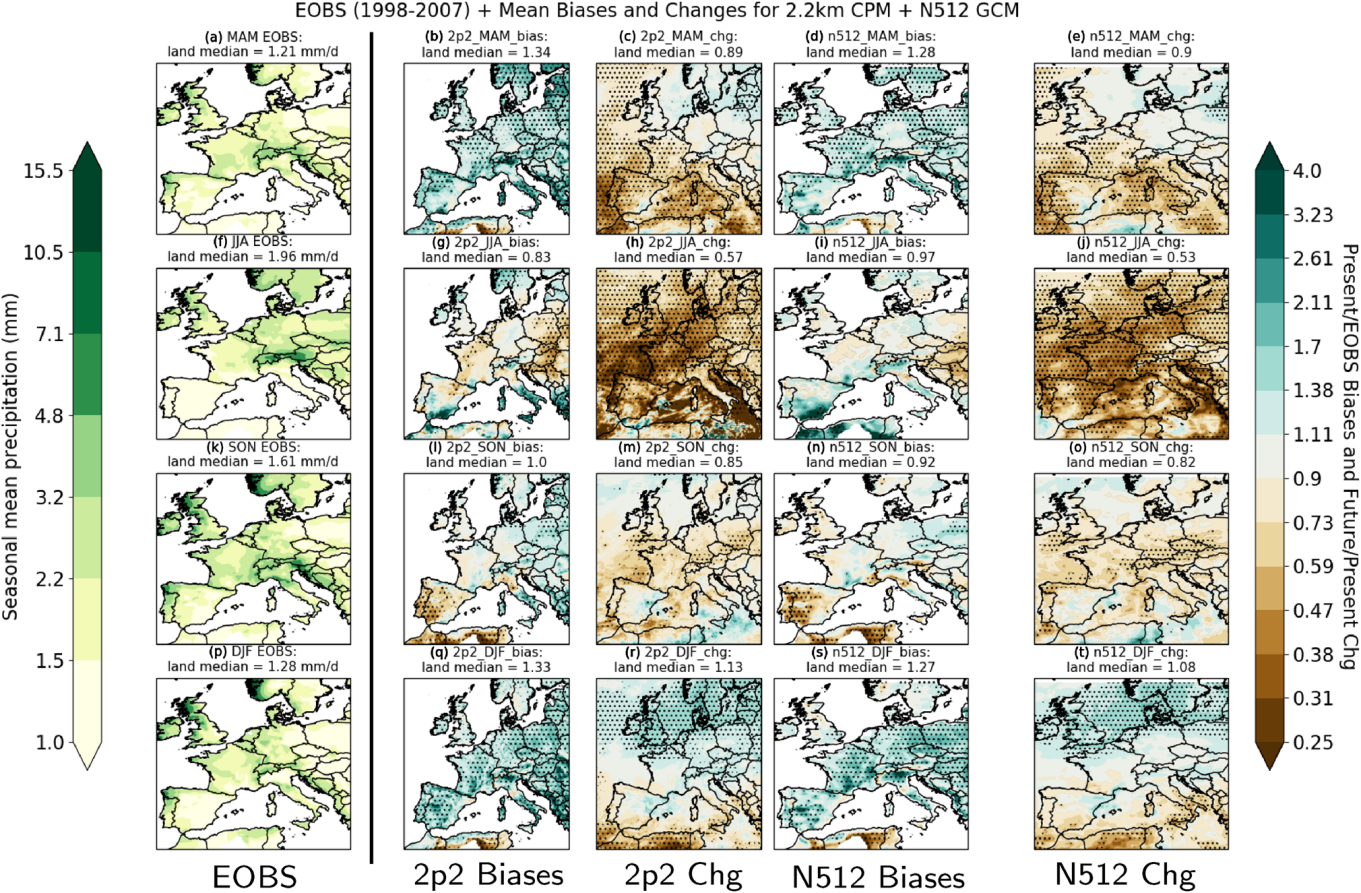
Seasonal mean precipitation biases (model divided by E-OBS) and future changes (future-climate simulation divided by present-climate simulation) for March–April–May (MAM; top row), June–July–August (JJA; second row), September–October–November (SON; third row), and December–January–February (DJF; bottom role) for the 2.2 km (“2p2”) and driving 25 km GCM (“N512”) simulations. The actual E-OBS observations are shown in the first column from the left. Statistical significance is determined by year-block bootstrapping (see Sect. 3.1) with multiple hypothesis testing* p* value adjustments (see Sect. 3.2; Wilks 2016); area with significant biases and changes are dotted. The median value over land grid points are given in each panel’s title

For the first time here, we examine convection-permitting model projected changes for a European-wide domain. This allows us to examine the influence of convection-permitting resolution for a range of different climates. We begin with an examination of the mean precipitation biases and changes. The 2.2 km CPM (“2p2”) and driving (“N512”) GCM seasonal mean precipitation biases and projections across Europe are shown in Fig. 2. Biases and changes that are significant at the 5% level are dotted.

### Mean biases

Seasonal mean model biases relative to E-OBS are shown in Fig. 2 (panels b, g, l, q) for the 2.2 km CPM and Fig. 2 (panels d, i, n, s) for the driving 25 km GCM. In brief, the 2.2 km CPM biases broadly follow the driving GCM biases with interesting summer (JJA) differences over Central and Eastern Europe.

**Fig. 3:**
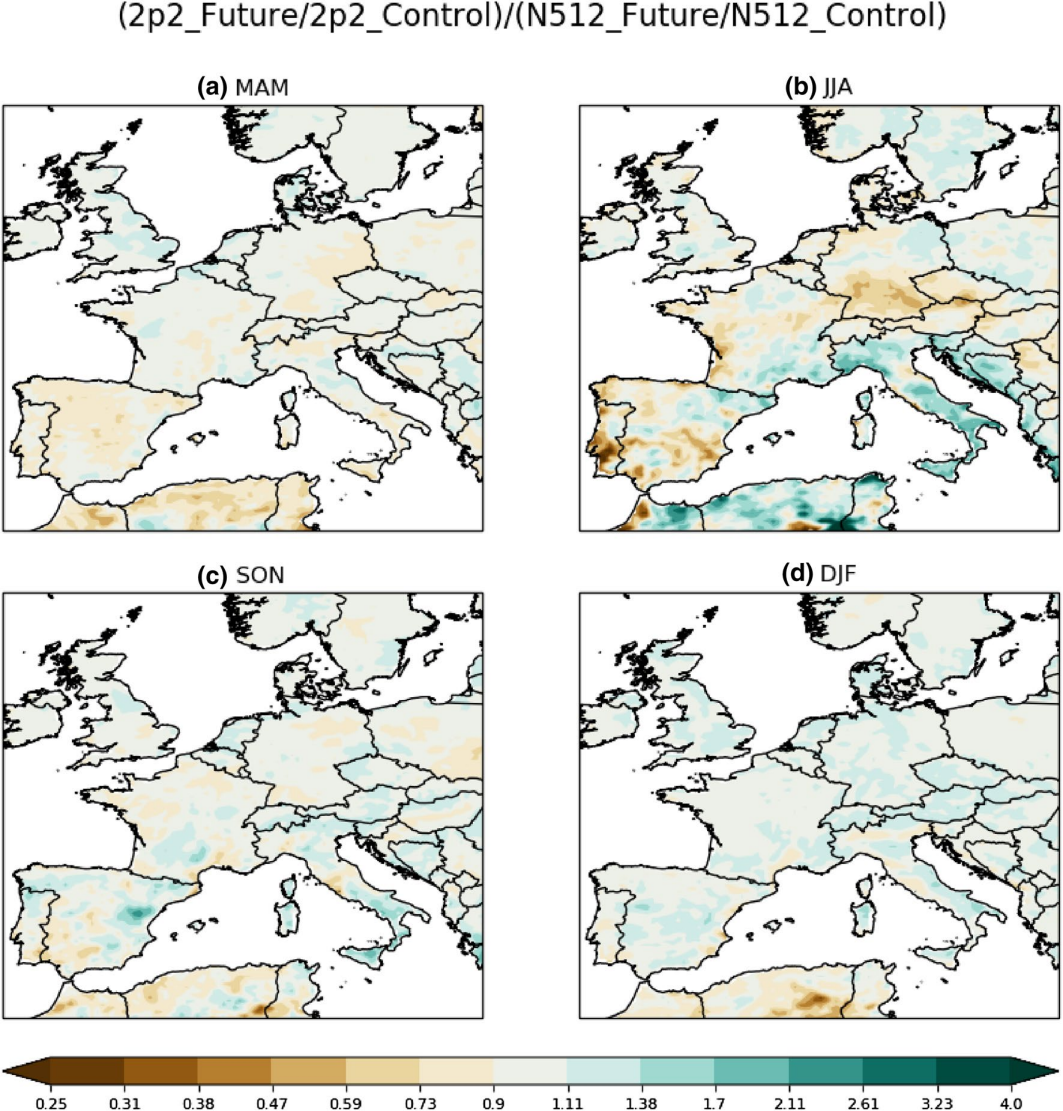
Differences in seasonal projected precipitation change between the 2.2 km and N512 simulations beginning for **a** March–April–May, **b** June–July–August, **c** September–October–November and **d** December–January–February. Data are first regridded to the common “N512” grid for comparison

Both models simulate excessive spring (MAM, panels b, d) and winter (DJF, panels q, s) precipitation. Over land, the models typically produce $30\%$ more precipitation than observations from E-OBS. Significant positive biases are not concentrated in specific areas, but are widespread across Europe from Iberia to Poland. Overall, the CPM biases for these two seasons follow the biases of the driving GCM.

Summer (JJA, panels g, i) precipitation biases are generally negative away from the Mediterranean. Although the GCM biases are generally not statistically significant, the CPM negative biases are somewhat larger and are significant over parts of Central and Eastern Europe (i.e. Poland, Belarus and Slovakia). In contrast, positive biases in summer mean precipitation are found over Scandinavia.

Autumn (SON, panels l, n) is perhaps the best simulated season in terms of mean precipitation biases. Biases over land are negligible. Although pockets of significant positive biases are found over Eastern Europe, they are small relative to biases in other seasons.

### Future summer European drying

The projected changes are shown in Fig. 2 panels c, h, m, r for the 2.2 km CPM and panels e, j, o and t for the driving 25 km GCM. The projected mean changes by 2.2 km and GCM simulations are dominated by a large ($50+ \%$) decrease in JJA mean precipitation (panels h, j) north of the Alps, with a smaller mean decrease in both spring (panels c, e) and autumn (panels m, o). Notable (10–$40\%$) and significant decreases in spring mean precipitation are found over France, Iberia and Italy. While autumn decreases cover a wide area across the northern half of the simulation domain, only the decreases (up to $\approx 40\%$) around the Alps, Czech Republic and Southern Poland are statistically significant. For the autumn Mediterranean wet season, the mean changes in Southern Europe are relatively moderate compared to the summer changes, with changes in neither season being statistically significant. Winter mean changes (panels r, t) are generally positive across Northern and Central Europe, but statistically significant changes exceeding $40\%$ are only found around the North and Baltic Seas. Broadly speaking, the CPM mean changes follow the driving GCM mean changes, akin to the CPM mean biases following the driving GCM mean biases.

**Fig. 4:**
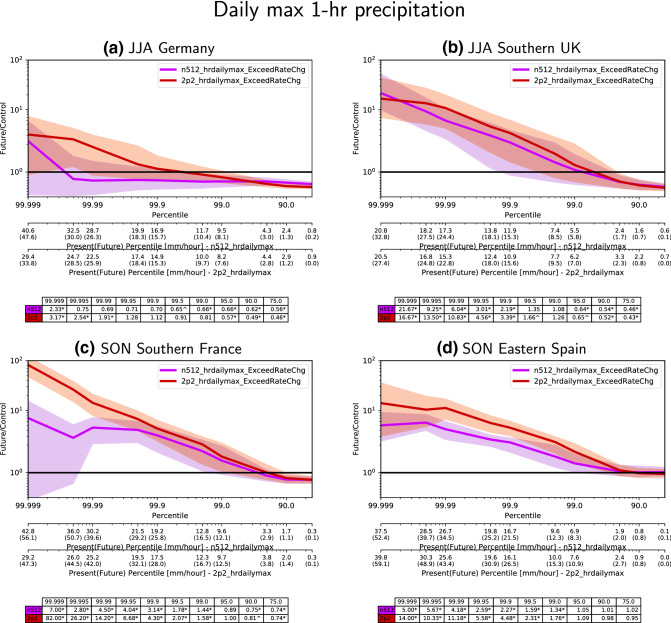
Future divided by present exceedance rates of present-climate thresholds for **a** JJA Germany, **b** JJA Southern UK, **c** SON Southern France, and **d** SON Eastern Spain at 25 km scale for the 2.2 km and N512 GCM simulations. Different simulations are marked with different colours—purple for the N512 GCM simulations, and red for the 2.2 km CPM simulations. The underlying present-climate thresholds for the two models are given in the additional x axes with the future-climate percentiles in parentheses. The actual exceedance change ratios are given by the table below. Year-block bootstrapping significance tests are applied (see Sect. 3.1), and future changes that are not rejected at the $5\%$ and $10\%$ level from the present-climate baseline are marked with “*” (asterisk/star) and “${\hat{~}}$” (caret) respectively

The summer drying signal largely comes from the driving GCM simulations, but there are differences between the CPM and GCM signal, may be due to their different precipitation physics and land surface feedbacks. We show the ratio of seasonal projected change between the 2.2 km and driving GCM simulations in Fig. 3. The 2.2 km CPM projects a more intense drying signal across Continental Europe north of the Alps in summer, but differences between the GCM and CPM projections are less clear for spring and autumn. As the 2.2 km CPM is known to have less frequent but more intense precipitation intensities (Berthou et al. 2018), the CPM JJA drying signal may be enhanced by drier soils due to the weaker penetration of high intensity precipitation; this may also explain the exacerbation of JJA mean dry biases inland as seen in Fig. 2g.

**Fig. 5:**
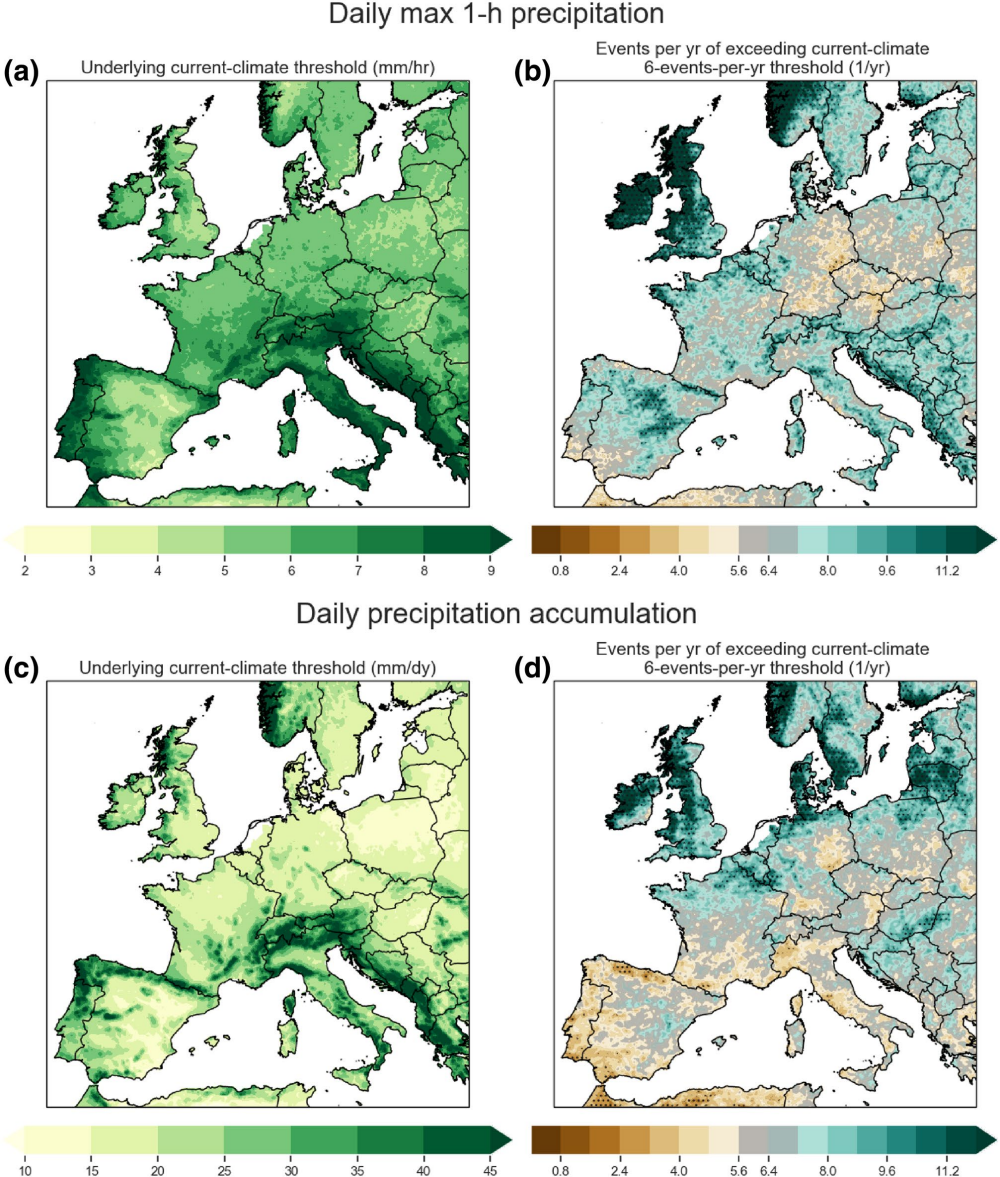
Future frequency of exceeding the 6th annual largest present-day daily maximum **b** 1-h and **d** 1-day precipitation intensity in the 2.2 km CPM. Right panels **b**, **d** are the actual future frequency (events per year), and left panels **a**, **c** are the underlying present-climate precipitation intensity thresholds in mm/hr and mm/dy respectively. By definition, the present-climate frequencies are fixed at 6 per year; hence any values above (green areas in the left panels) or below (brown areas in the left panels) 6 indicate increases and decreases respectively. Dotted areas are significant at the $5\%$ level after corrected with multiple-hypothesis testing (see Sect. 3.2 Wilks 2016) with uncorrected* p* values estimated using Z test assuming threshold exceedances as a Poisson process. Data are regridded to a common 12 km grid

The above projections are diagnosed from single-realization climate simulations. To put them within the context of large multi-model multi-ensemble projections for Europe, we now compare these with RCP8.5 projections from the 12 km Euro-CORDEX ensemble (Jacob et al. 2014) for the same time-period. Before the comparison, we note that the Euro-CORDEX ensemble is driven by the CMIP5^2^ 5 GCM simulations (Taylor et al. 2012), while our driving GCM simulations use prescribed SSTs (Mizielinski et al. 2014); hence, we do not expect our projections to be identical to the Euro-CORDEX ensemble. The Euro-CORDEX projections (Jacob et al. 2014) generally show an increase (decrease) of annual mean precipitation for Northwestern (Southern) Europe. For spring, the Euro-CORDEX ensemble mean shows an increase in northeast and a decrease in Southwestern Europe; the 2.2 km CPM projections project a somewhat similar spatial pattern but with a larger area showing decreases. Although the Euro-CORDEX ensemble mean indicates a substantial (5–25%) decreases in summer mean precipitation, the 2.2 km CPM projected decreases are often higher (i.e. $50+\%$ for parts of France and Germany). The Euro-CORDEX ensemble mean projected autumn changes show a mean increase (decrease) for Northeastern (Southwestern) Europe, while our simulations show a decrease across most of Northern and Central Europe up to $\approx 40\%$. For winter, the 2.2 km CPM projected increases across Northern Europe are generally consistent with the Euro-CORDEX ensemble mean.

### Percentile-based exceedances across Europe

Where long local time series are not available, regional frequency analysis is commonly used for extreme value analysis (RFA; Hosking and Wallis 1993). This has the advantage of replacing space for time therefore drawing from regional data pools. In RFA, regions are assumed to be homogeneous for precipitation extremes, and the pooled data samples independent of each other. A pooled percentile approach is used in Kendon et al. (2014) for SUK because it better accommodates precipitation data with different biases but may still get the relative ordering of the intensities correct (i.e. radar data may have negative biases at higher intensities (Harrison et al. 2000), but the relative ordering of precipitation intensities should still be approximately correct). As we are comparing models with different resolutions here, we first upscale all model data to a common 25 km GCM grid. We do not specifically account for spatial coherence (i.e. multi-grid point events) in this analysis, as is common practice in regional frequency analysis (Hosking and Wallis 1993).

To further ensure that results are comparable with previous ones (e.g. Chan et al. 2014a; Kendon et al. 2014), we also decluster the hourly data. The declustering approaches in Kendon et al. (2014) and Chan et al. (2014a) are different. The former considers continuous precipitation episodes, while the latter considers average separation times of peak precipitation (Ferro and Segers 2003). Here, we take an approach that is comparable but simpler than the latter approach and decluster hourly extremes by taking the daily maximum of hourly precipitation at each grid point, assuming the local daily maximums to be independent. The current method is straightforward to apply, transforms and simplifies hourly data into daily data, and includes low/no precipitation days in the analysis. All subsequent analysis with hourly precipitation uses the above approach.

The metric of interest here is the exceedance of current climate thresholds (Kendon et al. 2014): we determine the current-climate thresholds by taking percentiles of regionally pooled precipitation intensities from the present-climate simulation, and we then determine how often these thresholds are exceeded in the future-climate simulation. We examine four regions for two seasons: JJA for Germany and the Southern UK, and for SON for Southern France and Eastern Spain; they are all labeled in Fig. 1. To illustrate the projected future changes, the future divided by the present-climate exceedance frequencies are shown in Fig. 4. The underlying current-climate percentile thresholds are given on the two x-axes below each figure panel together with the future-climate percentiles in parentheses. The regions have been chosen due to their well-defined extreme precipitation season (see Sect. 4.5). Germany and the Southern UK have a well defined summer peak in hourly precipitation extremes; the two Western Mediterranean regions have a well defined autumn peak in hourly precipitation extremes.

**Fig. 6:**
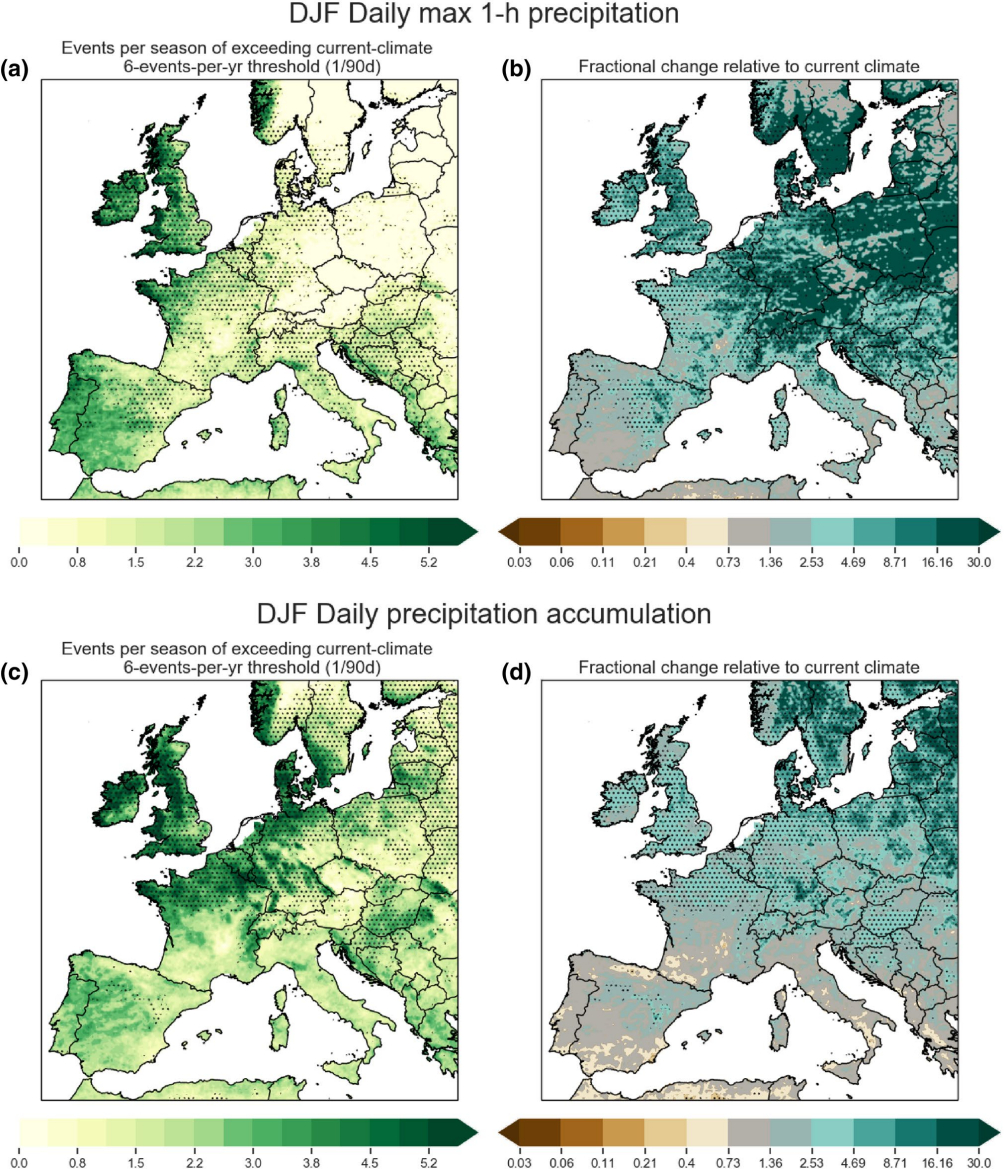
Similar to Fig. 5, but the annual threshold is applied to DJF only; hence, the present-climate frequency is no longer fixed and depends on event seasonality. The left panels **a**, **c** show the the actual future frequency (events per season; same as in **a**, **c** in Fig. 5), but the right panels **b**, **d** now show the future divided by present change. Note that left panel has different units (events per 90 days), contour interval and colour map than in Fig. 5. Dotted areas indicate changes that are significant at the $5\%$ level. Multiple-hypothesis testing corrections are applied

**Fig. 7:**
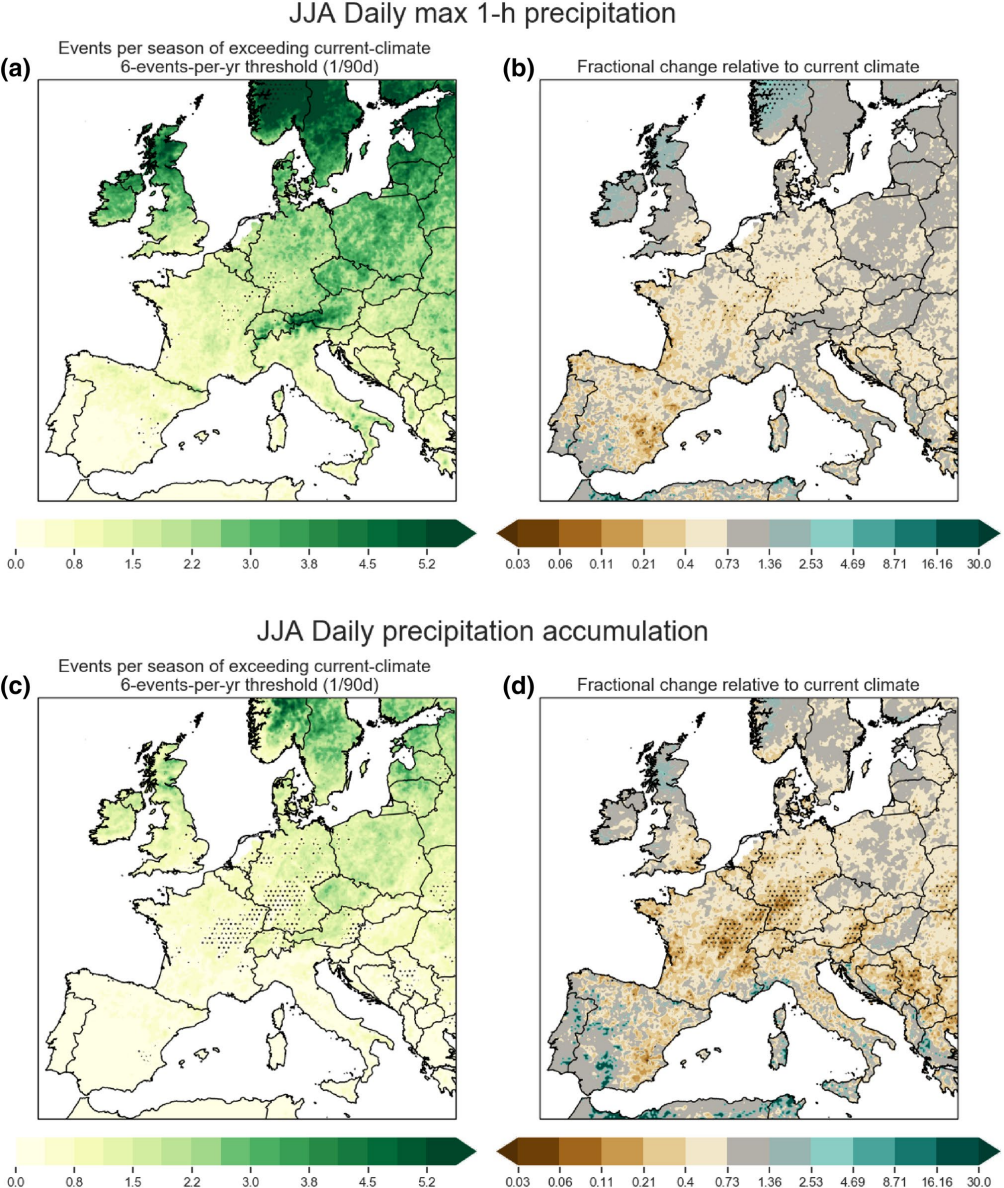
Same as in Fig. 6, but for JJA

The projected changes for each region differ substantially. Germany (panel a), where mean summer precipitation changes are strongly negative (Fig. 2), shows small to negligible increases for future hourly extremes; for the 2.2 km CPM projections, it is only at percentiles greater than 99.99 that we see any statistically significant ($3 \times$) increase in future exceedances. For Southern UK (panel b) where the projected decreases in mean summer precipitation are less severe than Germany, both the 2.2 km CPM and GCM project increases in exceedance frequencies for present-climate percentile thresholds above 99.0, with the CPM increases usually being somewhat higher. The 2.2 km CPM projections for the two Western Mediterranean regions (Southern France and Eastern Spain, panels c, d) for SON are clearly positive with 10 fold increases in events that exceed the present-climate 99.99 percentile. The GCM projected changes are positive as well, but do not see the same 10 fold increase for percentiles up to 99.999 (i.e. $2 \times$ to $8 \times$ increase).

**Fig. 8:**
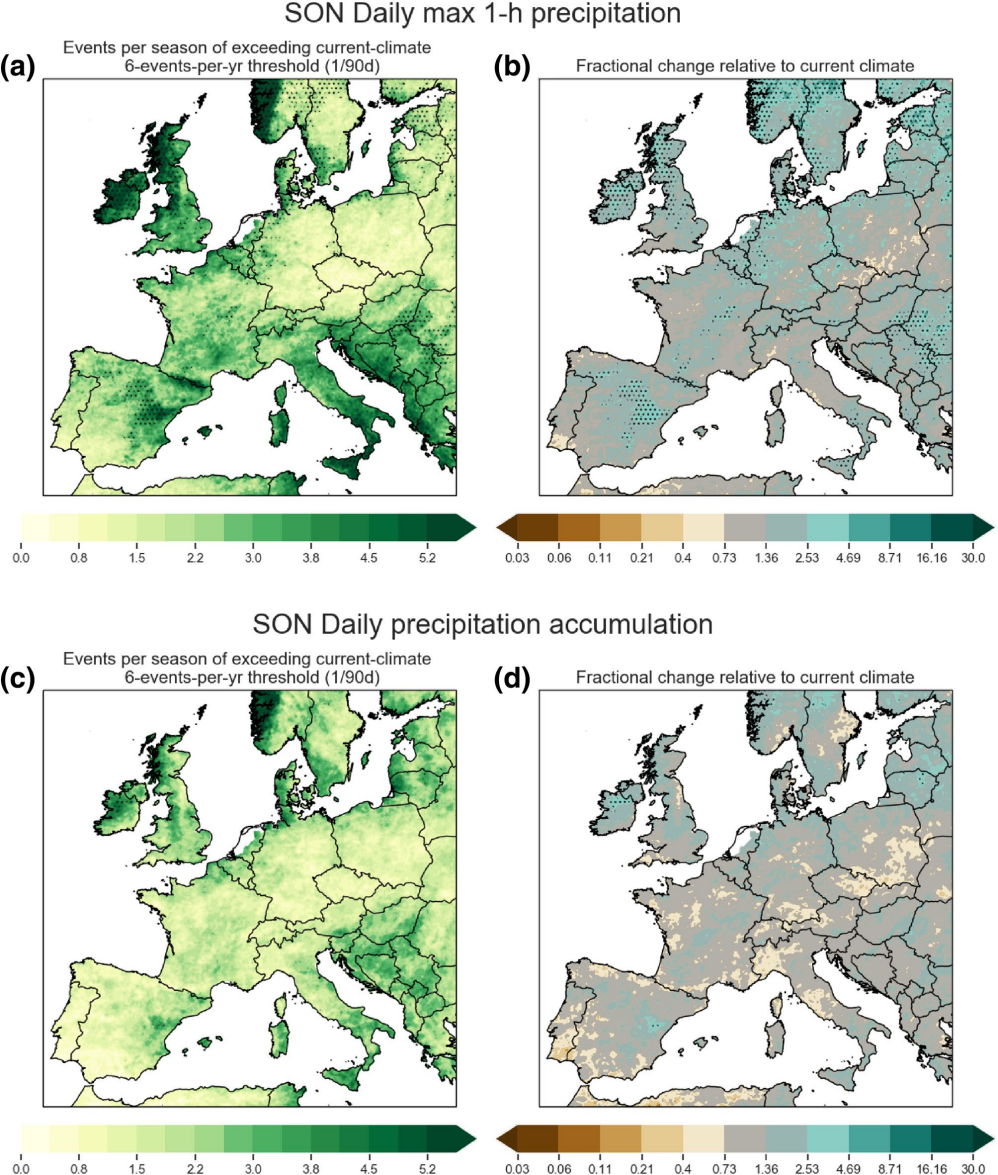
Same as in Fig. 6, but for SON

The future increase in exceedance frequencies occurs against the backdrop of intensity increases for the higher percentiles as indicated by the additional x-axes below each panel. With the exception of Germany, all three regions see intensity increases in hourly peak intensities for percentiles above 99.0 (corresponding to intensities of more than 6–10 mm/h, depending on the region). These projected increases are consistently higher for the CPM simulation. For Southern France, the CPM projected hourly peak intensity increase for the 99.999 percentile exceeds $50\%$. For Germany, increases begin at a higher percentile than the other three regions, but the increases start at a lower percentile for the CPM (99.9 percentile) than the driving GCM (99.999 percentile).

The underlying current-climate percentile thresholds are often higher in the lower-resolution GCM than the 2.2 km CPM. Since the analysis is conducted at the GCM horizontal scale, the regridded CPM intensities depend on the simulated spatial precipitation structure. We have explored this issue in further detail in the Supplementary Materials, especially how the spatial-averaging scale affects projected future Southern UK changes. In previous studies, areal precipitation averages from lower-resolution parametrised-convection models can indeed be higher than CPMs (Hohenegger et al. 2008; Lean et al. 2008) even if CPMs have a tendency to overestimate the intensity of the heaviest events (Kendon et al. 2012).

Overall, there are considerable differences between projections for Germany compared to the other three regions. Despite large regional variability in projected hourly intensity changes, both models project increases for the highest percentile ($\ge 99.995$) events for all four regions with the 2.2 km model projections being higher. Germany is an exception that the GCM projects a reduction of lower percentile events.

### Local projection of future extreme changes

Pooled regional analysis may increase the detectability of changes by increasing sample size, but this should ideally only be applied to homogeneous regions (Hosking and Wallis 1993). Formal definitions for homogeneous regions are often defined for point observations. Such an approach is difficult to apply to gridded climate model data due to the data’s high spatial density and the model’s ability to resolve detailed spatial inhomogeneity. Here we define our regions based on previous work (Kendon et al. 2014; Berthou et al. 2018), acknowledging that homogeneous regions are difficult to define precisely. Here we use the same approach as Chan et al. (2014b), and look at changes for each grid point of Europe. As sample sizes are now smaller and fixed locally,^3^ the robustness of these results is harder to establish.

Future annual frequencies from the present-climate simulation (future-divided-by-present) and the underlying precipitation thresholds are shown in Fig. 5 for daily maximum 1-h and daily precipitation. Note for the annual case, the change is just future-climate annual frequencies divided by $\frac{6}{360}$^4^. Significance is estimated by estimating the* p* value with block bootstrapping, with multiple-hypothesis testing adjustments (Sect. 3). The hourly thresholds (panel a) are typically between 5 and 10 mm/h, lying on the lower range of the thresholds examined in Sect. 4.3. The daily thresholds (panel b) are typically between 10 and 50 mm/day with the highest values over orographic regions (e.g. the Alps, Pyrenees, Basque Country, etc.).

**Fig. 9:**
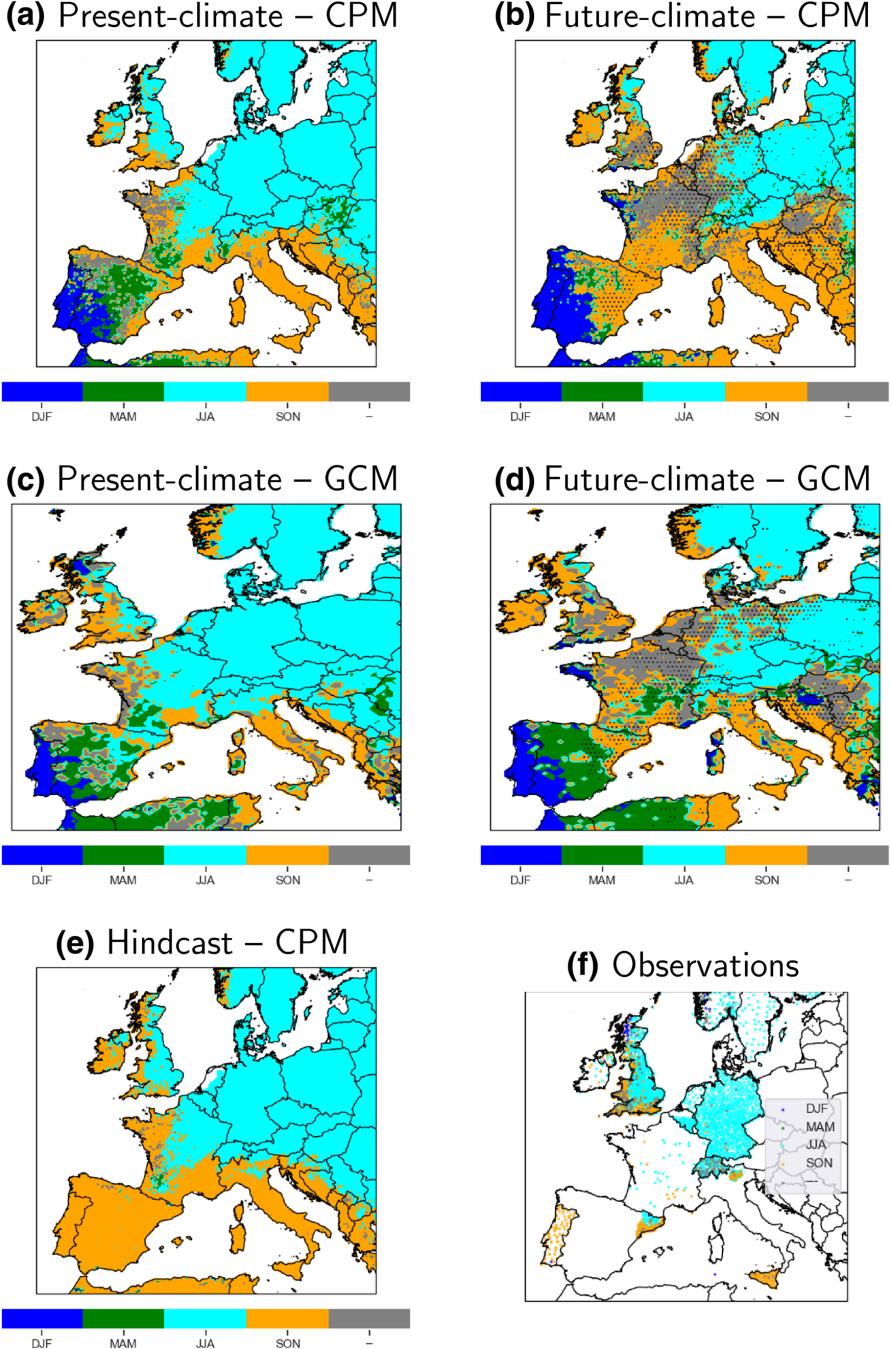
Season (MAM, JJA, SON, and DJF) with the most hourly extremes for the **a** present- and **b** future-climate 2.2 km CPM simulation, **c** the driving present- and **d** future-climate GCM simulation, **e** hindcast 2.2 km CPM simulation, and **f** GSDR station observations. Extremes are defined here as the heaviest 60 daily maximum 1h precipitation events throughout the 10-year simulation (equivalent to the 6 largest events per year on average, see Fig. 5). We greyed-out grid points and stations with seasonal differences that are not significant in a one-way $\chi ^{2}$ test at the $5\%$ level. For panels **b**, **d**, we dotted areas with significant future distribution changes (at $5\%$ level with the $\chi ^{2}$ test) relative to the present-climate simulation (see Sect. 3.4). Multiple-hypothesis* p* value adjustments (Wilks 2016) are applied to both the seasonal differences and their future change separately (see Sect. 3.2). Model sea grid points are masked for panel **a**–**e**

For daily maximum of hourly precipitation, grid points with significant increases in frequency are concentrated over Ireland, the UK and Norway (almost 2 times increase) with pockets of significant increases around the Mediterranean and over high orography (Alps and Pyrenees). Decreases are found in Central and Eastern Europe, but most of them are not statistically significant.

Like the extremes for daily maximum of hourly precipitation, daily frequency increases are also concentrated over Northern Europe. Larger areas of significant positive change are found not only over the UK and Norway, but also around the Baltic Sea and Denmark. Outside of Northern Europe, the only pockets of significant changes are found in Iberia/Morocco (negative) and Slovenia/Hungary (positive).

The annual threshold can also be applied seasonally to examine the seasonal distribution. We first concentrate on DJF here (Fig. 6) as it is the season that sees the most considerable change. In contrast with the annual changes, we find much larger areas of increasing frequency for both hourly and daily precipitation extremes; significant increases in frequency for hourly extremes are now found across the Northern Mediterranean, and significant increases for daily extremes are now found over a much broader area across Northern Europe. In fact, some areas in Central Europe see tenfold plus increases.

**Fig. 10:**
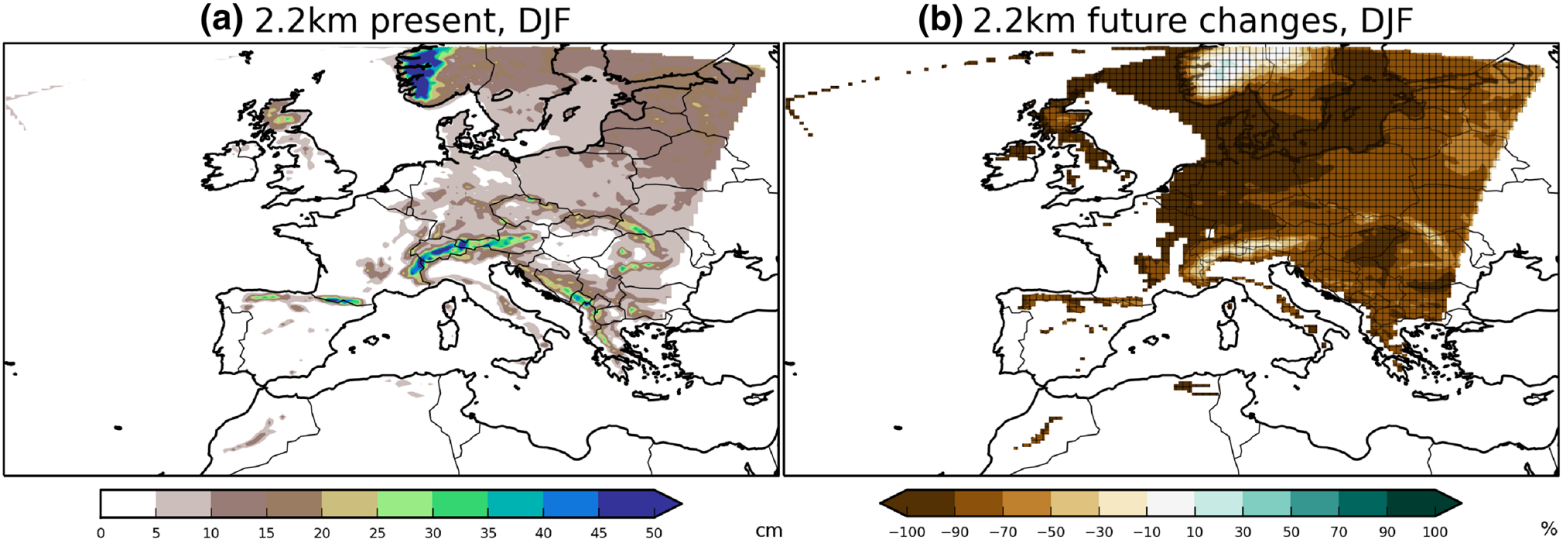
Shown in panel **a** is the 2.2 km present-climate simulation DJF snowfall (melted water equivalent), and its projected future $\%$ change are shown in panel **b**. Note very large decrease across lowlands across Central Europe

DJF is not the most important season for extreme precipitation under current climate conditions (see Sect. 4.5)—Northern and Southern European hourly precipitation extremes tend to occur in JJA or SON, especially extremes that are associated with deep convective storms. JJA and SON changes are shown in Figs. 7 and 8. For JJA, only Norway and the Western British Isles show exceedance frequency increases, and much of these are not statistically significant. In fact, exceedance frequency changes are mostly negative across Continental Europe with the largest negative changes in the frequency of daily extremes across France and Germany. SON exceedance frequency changes are mostly positive. Significant local increases to 1-h precipitation extremes are found mostly over Northern Europe, with local pockets around the Mediterranean. In SON exceedance frequency changes to daily extremes are locally statistically insignificant nearly everywhere.

Overall, results here and in the previous section indicates that the detection of changes in the frequency of heavy precipitation events often require a regional approach. Local grid point changes are difficult to diagnose and interpret unless the local signal is large (like increases over the UK and Norway).

### The changing seasonal cycle of extremes

Regardless of whether changes are diagnosed using pooled or individual grid point data, there is a clear pattern that non-summer hourly extreme precipitation changes are much larger than the summer changes across Europe. Summer and autumn show the largest frequency of extreme hourly precipitation in the present climate. The tendency for greater increases in the frequency of winter heavy precipitation (Fig. 6) suggests fundamental changes to the seasonal cycle of extreme events with warming. The combination of seasonal mean and extreme precipitation changes may have important implications for impacts. For instance, even though summer extreme changes are relatively small in intensity and frequency terms, the greater incidence of antecedent dry conditions may lead to much greater surface erosion; in contrast, an increase of winter extremes on top of already wetter winter conditions may lead to more winter flooding.

In Fig. 9, we show the peak seasons for 6 n daily maximum hourly precipitation events for the 2.2 km CPM simulations, 25 km GCM simulations, and GSDR observations. In both CPM and GCM present-climate simulations, events tend to peak in either summer (Northern Europe) or autumn (Southern Europe around the Mediterranean). Compared with the GSDR observations, the present-climate simulations capture the correct seasonal timing of hourly extremes over the UK (mostly JJA, some SON), the Low Countries (JJA), France (JJA), Germany (JJA), Scandinavia (JJA), Switzerland (JJA), coastal Catalonia (SON; see discussion below for inland), and Sicily (SON). A statistically significant shift away from a summer peak to either an autumn peak or no-peak-at-all is evident across France, the British Isles, the Low Countries, Spain, and the Northern Balkans in the future-climate simulation.

Most of Southern Europe sees relatively small changes in the timing of the seasonal peak with most extremes still occurring in autumn. There is however a significant shift in timing from a spring to an autumn peak over the interior of Iberia. The apparent winter-spring event peak over Western and Central Iberia in the present-climate 2.2 km simulation is not a feature found in the 2.2 km CPM hindcast (panel e). Instead, the winter-spring peak is a feature from the driving GCM simulation (panel c). This is also not found in the observations; for instance, Portuguese observations indicate hourly extremes are most common during autumn (Fig. 9f), but the 2.2 km and N512 present-climate simulations have an incorrect DJF peak. For inland Catalonia, none of model simulations (SON for both hindcast and present-climate simulations) agree with the observed GSDR (JJA) peak. The simulated Iberian extreme precipitation seasonality is wrong in the GCM and GCM-driven simulations, and results here are another example of the degree of control that the GCM lateral boundary conditions have over the downscaled 2.2 km CPM simulations. The same lateral boundary condition controls can be seen in the future changes as well, with the seasonal shifts in the driving GCM (panel d) generally similar to the downscaled CPM.

The shifting of extremes away from summer plus the large increase in both winter mean and extreme precipitation lead to the important question of future snowfall changes. Large changes to the ratio between snowfall and rainfall can have large implications for both social-economic impacts and regional climate feedbacks. The 2.2 km projected DJF mean melted-water-equivalent snowfall changes are shown in Fig. 10. There are widespread large decreases in snowfall across the Central/Eastern Europe lowlands and Scotland. Over Germany, the Low Countries, and Southern Scandinavia, the decrease approaches $100\%$. Lower decreases and even some increases are projected over high Alpine and Norwegian orography. Our snowfall projections over the Alps are somewhat more moderate than the $45\%$ decrease projected in Frei et al. (2018); however, their projections were based on lower-resolution climate models, so differences may be down to the representation of orography, otherwise the lowland snowfall projections are qualitatively similar.

### Within the context of previous UKMO CPM simulations

The simulations presented here are different in many ways from the previous UKMO CPM simulations (Chan et al. 2013, 2014a, b; Kendon et al. 2012, 2014): different model configurations and versions, different lateral boundary conditions, and different model domains. Therefore, there is no straightforward way to attribute the differences between the new and previous modeling results. Nevertheless, here we document the differences, so that impact studies using the previous simulations (Dale et al. 2015; Ockenden et al. 2016) may understand how the model results are evolving which may then affect the interpretation of their results.

Further details of the comparisons can be found in the Supplementary Materials. To quickly summarize the key results:

1. Both the previous 1.5 km simulations and the newer 2.2 km simulations do not have major mean precipitation biases over the Southern UK (Supplementary Fig. 1).
2. The new projections show higher frequency increases in winter mean precipitation but a more severe summer drying (Supplementary Figs. 2, 3).
3. The new 2.2 km CPM projections show larger frequency increases than the previous 1.5 km CPM projections in summer hourly extremes (Supplementary Figs. 7–10).
4. The projections of change to extreme precipitation are dependent on the spatial-averaging scale (Supplementary Figs. 11–12) with the 2.2 km CPM projections showing larger increases at the GCM scale compared to the native grid scale.
5. We have explored how sensitive the spatially-pooled results are to spatially-correlated events like “grid point storms” that are found in some of our “grey-zone” resolution simulations (Supplementary Section 2).

## Conclusions and discussions

This is the first time that a CPM has been used to provide Europe-wide future-climate projections. We find projections over Europe are dominated by wetter winters across Northern Europe with increased extremes. Summer is drier across Northern and Central Europe; the decrease in mean precipitation over France and Germany is even more severe than for the UK. Projected summer hourly extreme changes are much more muted across Continental Europe as shown in Figs. 4 and 5; there is even suggestion that summer hourly extremes will become less frequent for Central Europe, but this result is not significant compared to year-to-year variability. While there is a decrease in mean precipitation for autumn, extremes are expected to become more frequent for some regions in Southern Europe (Fig. 4). Importantly, we find the 2.2 km CPM hourly extreme precipitation projections are consistently higher than the driving GCM for all examined regions (UK, Germany, Southern France, Eastern Spain), and statistically significantly so in the Mediterranean for “very high” ($99.9+$) percentiles; for instance, our simulations project five fold plus increases in 20 + mm/h events at the 25 km scale for the Southern UK, Southern France and Eastern Spain (Fig. 4). Otherwise, the GCM and CPM results look broadly similar in terms of mean precipitation and seasonality changes.

The relatively muted summer changes to extreme precipitation are in contrast with the much larger winter increase in extremes—a consistent result from the previous 1.5/12 km model projections which were performed over much smaller domains. This leads to significant changes to the future seasonality of hourly extremes across Northern Europe where such changes may have extremely important physical and social-economic implications. The larger winter increase is generally consistent with temperature-precipitation extreme scaling which favors higher scaling rates at lower air temperatures (i.e. $\lesssim 20\,^{\circ }{\text {C}}$) (Hardwick Jones et al. 2010; Utsumi et al. 2011).

The winter projections show increases in extreme and mean precipitation yet decreases in snowfall. Hence, the projections imply fundamental changes to future winter weather and climate risks—such as reduction of snow traffic disruptions and spring snow-melt flooding, the need for additional management for increased winter water and hydro power capacity with offsets to higher summer demands due to warming and drying and heightened risks for rainfall-driven winter (flash) floods (Frei et al. 2018).

Two different approaches are used to estimate our projections—projection per pooled region and projections per grid point. Projecting local (grid point) changes are heavily limited by the shorter time series available at each grid point; in contrast, pooled analysis (e.g. Fig. 4) significantly increases the sample size and permits the examination of the far tail of the extremes, but can over-count the same event multiple times. The over-counting of hourly events in regional pooling analysis is reduced by using the daily hourly maximum precipitation in this study as data are temporally declustered, but this still does not account for spatial coherence. Precipitation is clearly a spatially and temporally correlated quantity, but the actual correlation (i.e. over-counting) is difficult to account for since many physical mechanisms can be involved, i.e. extreme events can be localized convection or organized convection/frontal precipitation that is organized at the synoptic scale. As we have demonstrated in the Supplementary Material section 2, spatial over-counting plus the possibility of very rare events can occasionally lead to large changes for the regional pooling results in which the spatial signature of the rare event is exacerbated. Hence, caution should be exercised when comparing changes for pooled regions and individual grid points; the latter is not subject to the above problem, where the impact of such very rare events is confined to the top-most extreme intensities at that grid point. That said, such pooling is common practice as long as the pooling is applied to a relatively uniform region (regional frequency analysis; Hosking and Wallis 1993). Individual grid point projections are not impacted by spatial correlation, but we need to account for the impact from conducting hypothesis tests together over many grid points (Wilks 2016). Results here demonstrate the type of differences when both approaches are applied—significant changes are much harder to obtain at individual grid points, and local changes for the highest extremes require extrapolations using extreme-value theory (Coles 2001) and are highly uncertain; instead, changes over a wide area can be examined. While individual grid point changes may not be statistically significant, they are consistent with the regionally pooled results. Future analyses may incorporate machine learning and pattern recognition methods where both temporal and spatial correlation of extremes can be accounted for Prein et al. (2017a).

Overall, the most important difference between the GCM- and CPM-projected changes of precipitation are in the metrics that are related to extreme precipitation intensity changes—either the actual intensities themselves or how often they are exceeded in the future-climate simulation (Fig. 4). The mean changes and timing of precipitation peak intensities appear to mostly controlled by the driving GCM; i.e. changes to the storm track and synoptic transients. There are hints for inland Europe that the CPM mean precipitation decreases for summer are somewhat more severe, but the sign of the change is already clearly evident in the driving GCM projections.

Current projection uncertainties are still based on only a 10-year single-realization for one specific (RCP8.5) emissions scenario and one specific time-period (end of 21st century). We mimic inter-annual variability via year-block bootstrapping, but this assumes that model years are reasonably representative of the greater (and unknown) probability distribution of model years which is untrue as decadal variability has been ignored. This can only be addressed with longer and/or multi-ensemble continental-scale CPM simulations, which is currently infeasible without incurring unreasonable computational costs. RCP8.5 represents the highest Intergovernmental Panel on Climate Change (IPCC) emission scenario, and CORDEX guidelines (Giorgi et al. 2009) recommend simulations for all RCP emission scenarios. Multi-model ensemble assessments are cornerstones to modern climate and weather sciences, and are recommended by the IPCC for climate change projections (Alley et al. 2007). For the UK, uncertainty in CPM projections can now be examined using the UKCP18 Local ensemble simulations (UKCP Project Team 2017), which were released in September 2019; headline results are now available in the official report (Kendon et al. 2019), which is available at https://www.metoffice.gov.uk/research/approach/collaboration/ukcp/guidance-science-reports. Beyond the UK, multi-model CPM projections over parts of Continental Europe are planned as part of the CORDEX Flagship Pilot Studies (Coppola et al. 2018) and European Climate Prediction System (EUCP; Hewitt and Lowe 2018) initiative. CPM projections for all of Europe are still perhaps too computationally expensive; each of our single 2.2 km CPM simulations required more than 1 year to complete with UKMO computing facilities. The future of CPM climate simulations is not just a scientific challenge but a technical one as well.

## Electronic supplementary material

Below is the link to the electronic supplementary material.

Supplementary material 1 (pdf 5338 KB)

## Footnotes

1 Some Italian stations have data as far back in the 1920s.

2 Coupled Model Intercomparison Project.

3 The sample sizes for pooled regions depend on the number of grid points included in the region.

4 6 days divided by 360 days; both present- and future-climate simulations use a 360-day calendar
