# Supplementary material for: Europe-wide precipitation projections at convection permitting scale with the Unified Model
Source: Clim Dyn. 2020 Jun 25;55(3):409–28. doi: 10.1007/s00382-020-05192-8 (PMC7370986; doi:10.1007/s00382-020-05192-8)
Supplement: Supplementary file 1 — Supplementary material 1 (pdf 5338 KB) [file 382_2020_5192_MOESM1_ESM.pdf]

Climate Dynamics manuscript No.
(will be inserted by the editor)

Europe-wide precipitation projections at convection permitting scale with the Unified Model – Supplementary Materials

Steven C Chan · Elizabeth J Kendon · Ségolène Berthou · Giorgia Fosser · Elizabeth Lewis · Hayley J Fowler

Received: date / Accepted: date

1 Comparison of new Europe-wide 2.2km and previous UK 1.5km CPM simulations

Here we document how the new 2.2km CPM results differ from previous UK-only CPM simulations at 1.5km resolution [Chan et al, 2014a; Kendon et al, 2012, 2014]. The original CPM simulations have different lateral boundary conditions and are based on an older generation of the UKMO Unified Model. The previous simulations are listed in Supplementary Table 1; these include not just the 1.5km simulations themselves but also a series of 12km RCM and 60km GCM simulations. All of them are compared here. We analyzed the same model years as in the main text (present: 1999–2008; future: 2099–2108).

The comparison between the 2.2km and 1.5km CPM projections comes with a major caveat that they are not independent from driving GCM differences. Thus, we discuss the projection differences in the context of how much they may be related to differences resulting from the underlying driving GCM simulations. Is CPM added-value sensitive to differences in the driving data?

Steven C. Chan
School of Civil Engineering and Geosciences, Newcastle University, Newcastle upon Tyne, UK.
(*visiting scientist at the Met Office Hadley Centre)
https://orcid.org/0000-0001-7695-3754
Tel.: +44-1392-884802
Fax: +44-1392-885681
E-mail: steven.chan@metoffice.gov.uk

Elizabeth J. Kendon
Met Office Hadley Centre, Exeter, UK.

Ségolène Berthou
Met Office Hadley Centre, Exeter, UK.

Giorgia Fosser
Met Office Hadley Centre, Exeter, UK.

Elizabeth Lewis
School of Engineering, Newcastle University, Newcastle upon Tyne, UK.

Hayley J. Fowler
School of Civil Engineering and Geosciences, Newcastle University, Newcastle upon Tyne, UK.

1.1 Mean and extreme precipitation biases in the hindcast simulations

Here we focus on the SUK biases between the two CPM hindcasts. NUK is not examined here as we do not have a 1.5km hindcast simulation over that domain.

The JJA and DJF differences between the 1.5km and 2.2km hindcasts over SUK are shown in Supplementary Fig. 1. Generally speaking, JJA mean precipitation is higher for the 1.5km hindcast than the 2.2km hindcast simulation, and this results in an overall positive (negative) bias for the 1.5km (2.2km) CPM hindcast across SUK relative to the UK5 observations. In the 1.5km CPM hindcast, the wet JJA biases are concentrated over South-east England – a feature that is absent in the 2.2km hindcast.

DJF precipitation totals are generally lower in the 1.5km simulation than the 2.2km simulation, but both hindcasts have more precipitation than the UK5 observations with positive biases in Eastern England. The 1.5km CPM hindcast has negative biases over Wales. Overall, the JJA and DJF seasonal means for both hindcast simulations are quite comparable, but there are perhaps some hints that DJF precipitation biases over Wales are lower in the 2.2km hindcast. The larger negative biases in 1.5km hindcast are consistent with the simulation domain being small and Wales being close to the lateral boundaries. These negative biases are absent from the 2.2km European hindcast as the small simulation domain issue is mitigated.

Overall, the above shows that the lower-resolution (2.2km) model has smaller biases than the higher-resolution (1.5km) one due to the updated model physics and/or larger domain size. A similar result is found in Fosser et al [submitted] with smaller UK-only CPM simulations in which increased resolution does not reduce the bias.

1.2 UK mean precipitation changes

Moving onto the GCM-driven future projections, we will include comparisons for NUK due to availability of GCM-driven 1.5km simulation over NUK [Chan et al, 2018]. The 1.5km and 2.2km CPM-simulated JJA and DJF seasonal means for SUK and NUK are shown in Supplementary Figs. 2 and 3. The two present-climate JJA simulations (panels b and d of both figures) are more-or-less comparable for both the NUK and SUK. Over SUK, the 2.2km present-climate simulation is drier with the 2.2km simulation having less precipitation than the 1.5km simulation over the southern half of SUK. The opposite is true for NUK, where the 1.5km present-climate simulation is slightly drier with differences in the southwestern part of NUK.

Future JJA projections (Supplementary Figs. 2 and 3, panels c and e) are dominated by a 35-to-55 % decrease in mean precipitation for both SUK and NUK. Over SUK, the 2.2km future-climate simulation shows a greater fractional decrease than the 1.5km future-climate simulation (Supplementary Fig. 2, panels c and e). The NUK JJA projections are closer between the 2.2km and 1.5km simulations, and the decrease is actually smaller for the 2.2km simulations (Supplementary Fig. 3, panels c and e).

For DJF changes over SUK, both present-climate simulations (Supplementary Fig. 2, panels g and i) are more-or-less comparable. The 2.2km present-climate simulation has less negative biases than the 1.5km simulation over the western half of SUK (i.e. Wales) – a feature that we also find in the hindcast simulations (Supplementary Fig. 1, panels e and f). For NUK, the 1.5km present-climate simulation is noticeably drier than the 2.2km simulation, and the differences are again mostly due to differences in the western half of the domain. On first sight, results suggest that the 1.5km CPM is underestimating orographic and coastal

precipitation, but this may also be a consequence of the simulation domain being too small and the orography being too close to the lateral boundaries.

Future DJF projections are shown in panels h and j. The future mean increases are much larger in the 2.2km simulations than the 1.5km simulations. The 2.2km projected median increases are 48% and 42% for SUK and NUK respectively, which are 2× and 3× larger than the 23% and 13% increase for the 1.5km simulations. Hence, the new 2.2km model projections are a significant upward revision from the ones that we have presented in Chan et al [2018].

One might suspect large winter mean precipitation differences are related to representation of the storm track, which is a defining difference between the two driving GCMs. The question is then whether the DJF differences between two CPM simulations are related to the differences in the driving GCMs or to structural differences between the two models or a combination of both. The DJF differences in the present-climate 60km "N216" GCM simulations and their projections are shown in Supplementary Fig. 4, and we compare this with Fig. 2 in the main text. For the present-climate simulations, the two GCMs appear to be showing the same differences that we have found between the two CPMs: the "N216" present-climate simulation is noticeably drier in the western half of UK. Thus the precipitation differences are likely to be due, at least in part, to the GCM stormtrack/circulation differences. The higher 2.2km CPM projected changes are consistent with the higher increases in the 25km "N512" future-climate simulation especially around the North Sea. Overall, the DJF differences over orography between two CPM simulations seem to reflect the GCM differences, in which the 25km "N512" GCM simulation is wetter for the western half of the British Isles. The influence of the JJA differences between the GCMs (Supplementary Fig. 4) is less clear; for instance, the 25km "N512" GCM present-climate simulation has a wetter Southern England than the "N216" GCM, but the differences between the 1.5 and 2.2km CPM present-climate simulations are actually opposite (i.e. 1.5km present-climate simulation is wetter than the 2.2km present-climate simulation; Supplementary Fig. 2, panels b and d).

1.3 Probability densities of UK hourly precipitation

Mean changes do not necessarily reflect changes at the tail of the probability distribution (i.e. "extremes"). Extreme hourly precipitation events may be negligible in terms of their contribution to the long-term seasonal mean, and reflect different kind of hydroclimate risks (such as "rapid" flash flooding instead of droughts and "slow" flooding). Previous UK CPM hourly precipitation analyses with the 1.5km CPM simulations have benefited from the availability of UK hourly radar observations [Harrison et al, 2000; Kendon et al, 2010]. We extend the analysis of the new 2.2km simulations with an additional new CEH-GEAR1hr product [Lewis et al, 2018]. CEH-GEAR1hr may provide a more reliable estimate than the radar observations in regions of poor spatial coverage such as Scotland [Chan et al, 2018].

1.3.1 Frequencies of specific precipitation intensities

To further explore the 1.5km and 2.2km model differences (especially ones from JJA), we examine the JJA and DJF hourly precipitation histograms for the hindcast and present-climate simulations in Supplementary Fig. 5[1]. Data are regridded to a common 12km grid,

[1] This is the same analysis as in Fig. 6 in Chan et al [2018].

and results from the additional UM10.1 simulations [see Supplementary Table 2; Fosser et al, submitted] are included. The histogram probability frequencies (y-axis) go as low as $\sim 10^{-6}$ (about $\approx$ 20-some events).

For JJA, all CPM simulations are overestimating (underestimating) the frequencies of 5-20 (≤ 2) mm/hr events as shown in Berthou et al [2018]. The 1.5km CPM simulations have more frequent "high" (10+ mm/hr) intensity precipitation than the 2.2km CPM simulations, and the 2.2km model-simulated frequencies are more consistent with the CEH-GEAR1hr (but not radar) estimates. In contrast in DJF, the higher 1.5km extreme intensities actually makes the 1.5km CPM simulations more consistent with the observations. All simulations fail to capture any DJF 20+ mm/hr events that are observed in both radar and CEH-GEAR1hr.

One would naively link larger CPM grid boxes with higher precipitation intensities, as moist convective ascent is under-resolved by the lower-resolution model [Hanley et al, 2015]. If this hypothesis is valid, the 2.2km European hindcast simulation would have higher extreme precipitation intensities than the 1.5km SUK hindcast simulation, which is not true. The two model simulations have different domain and model physics (see Sec. 2), and such differences are likely to have an effect on their simulated precipitation intensities. The hypothesis does appear to hold for the "additional" UK 1.5km and 2.2km hindcast simulations for bins above 10 mm/hr. In this case, the model physics and simulation domain are the same, hence this is a fairer test of the role of resolution on precipitation intensities. Both "additional" UK hindcasts have lower frequencies in the high intensity bins than the original 1.5km SUK hindcast simulation, suggesting that the 1.5km SUK hindcast is the one that stands out from the rest.

Due to the lack of 1.5km hindcast simulation for the NUK, the comparisons above are limited to the 1.5km NUK present-climate simulation (Supplementary Fig. 6). NUK winter results are generally similar to SUK with models showing similar biases. For JJA, we see smaller differences between the 1.5km NUK and 2.2km European present-climate simulations for the 10+ mm/hr bins.

The projected JJA/DJF SUK probability distribution changes for the 1.5km SUK and 2.2km European simulations are shown in Supplementary Fig. 7. Future JJA changes for both simulations are dominated by frequency decreases for bins between 0.1 and 10 mm/hr and frequency increases for bins above 10 mm/hr and below 0.1 mm/hr. The frequency decreases for bins between 0.1 and 5 mm/hr are especially severe ($50+\%$ decrease) and are consistent with the mean changes (Supplementary Fig. 2). In contrast, the increases for 10-50 mm/hr bins are higher in the 2.2km simulation; for instance, the 2.2km projected increase for 20-50 mm/hr is $3\times$ higher than the 1.5km model projections.

DJF changes (Supplementary Fig. 7, panel b) have even higher frequency increases than JJA for the 10+ mm/hr bins, yet are without the large negative changes in the lower intensity bins. The DJF increases for the 10-20 mm/hr bins are $4\times$ larger than the JJA changes; however, the actual future frequencies are still lower than the JJA frequencies. For the 10-20 mm/hr bin, the 2.2km model projections are about $2\times$ higher than 1.5km model projections. The same analysis results for NUK are shown in the Supplementary Fig. 8. Much of the results are qualitatively similar to the SUK, but NUK has higher (lower) JJA (DJF) frequency increases in hourly extremes (10+ mm/hr) than the SUK. Like SUK, the frequency increases for the higher intensity bins are generally higher for the 2.2km simulations with the exception of the DJF 20-50 mm/hr bin.

Similar to the mean precipitation changes (Supplementary Sec. 1.2), the above projection differences are caused by a combination of model physics, domain size, and driving condition differences. However, both sets of CPM simulations project future increases in

JJA and DJF hourly intensities above 10 mm/hr; they only disagree in the magnitude of the change. The 1.5km SUK hindcast and present-climate simulations have more frequent high intensity precipitation than the 2.2km hindcast and present-climate simulations; yet, the 1.5km model projected increases are actually lower than the 2.2km simulations for such intensities.

1.3.2 Percentile-based comparisons and exceedance of current extreme thresholds

Here we conduct the same spatial pooling analysis for SUK as we performed in main text. Results are shown in Supplementary Fig. 9, and the 1.5km and 12km simulations are included.

For the present-climate simulations, the 1.5km CPM simulation generally has the highest percentile values except at the highest percentiles (> 99.99) where the 12km simulations overtakes the 1.5km simulation. For percentiles below 99, the 2.2km present-climate simulation is generally comparable with the 1.5km simulation, but the 2.2km CPM intensities grow at a slower rate than the 1.5km CPM ones at higher percentiles.

For the future projections, there is a general increase in both future percentiles (panel b) and exceedance rates (panel c) for percentiles greater than 99. The only exception is for the 1.5km CPM projections for which changes above the 99.0th percentile are essentially zero. For percentiles below 99, all model future projections are negative.

The large 12km model projection uncertainties stand out from the rest of the model projections. Even though the 12km simulations are an intermediate model between the driving GCM data and the 1.5km CPM simulations, there are large differences in their projected percentile and exceedance rate changes for percentiles greater than 99. This is traced to an extreme outlier occurring on 2 days of the simulation (see Supplementary Section 2). There are much smaller differences between the 2.2km and 25km "N512" model results, in which projected changes between the 2.2km and 25km simulations are generally not statistically significant (except for the 99.9 and 99.95 percentiles); however, we note that for percentiles from 99 up to 99.99, the 2.2km projected increases are consistently higher than the driving 25km "N512" GCM.

In Kendon et al [2012] and Chan et al [2014b], we found large differences in extremes between the 1.5km and 12km simulations based on data regridded to a common 12km grid. In Chan et al [2014b], the 12km simulation is prone to large "grid point storms" (for example, Williamson [2013]) which cause large shape biases to its probability distribution. We do see large differences here again at the GCM 25km horizontal scale, but results here also appear in contradiction with the ones in Kendon et al [2014] and Chan et al [2014a], where we found a robust increase for 1.5km hourly precipitation extremes at the 12km horizontal scale. Repeating the above analysis using data regridded to 12km (Supplementary Fig. 10), we obtain results similar to Kendon et al [2014] with significant increases in 1.5km model future extremes[2]. The 12km model future-climate projections at 12km scale are somewhat higher than the changes at 25km scale, and are similar to the 1.5km model projections analyzed at 12km; this last result appears to contradict with ones shown in Kendon et al [2014], but the 12km projections have much higher uncertainties than the CPM projections. On the removal of a single 2-day event, the 12km aerosol-physics-enabled model results of Kendon et al [2014] are regained. This indicates that the projected changes for the 12km simulations

[2] For instance, both Kendon et al [2014], and the analysis here found the 99.999 percentile exceedance rate increase to be ≈ 5.0 despite different data sampling approaches and different model simulations.

are highly sensitive to the random sampling of grid point storms in the simulation, and highlights the lack of robustness of any projected change to precipitation extremes in this model (see Supplementary Section 2 below).

The question remains why the 1.5km model projections are so different than the 2.2km model projections. Clearly, the 1.5km model is driven by a different GCM and it has many fundamental differences with the 2.2km model – a different simulation domain size and even different model physics.

Our results suggest that the model projections are horizontal scale dependent. At the 25km "N512" GCM scale, the 25km "N512" GCM projection imposes its own baseline, and we should expect the 2.2km model projections should be somewhat consistent with the driving GCM at the driving GCM scale. However, some differences should still be expected as small scale CPM features are unlikely to upscale exactly; this is hinted at by the 2.2km projections for moderate/heavy hourly precipitation frequency being consistently higher than the driving GCM projections. The GCM simulations can only simulate precipitation systems of scales larger than the GCM resolution. Smaller precipitation systems simulated by the higher resolution CPMs at sub-GCM scale are a result of fundamentally different model physics, have their own spatial structure and dynamics, hence may have a different future projected change. The departure of the 2.2km CPM projection at the 12km scale from that at the 25km scale is actually added value for the 2.2km CPM. As the 1.5km simulations are nested through a 12km simulation, the influences from the driving GCMs are indirect. Analysing the 1.5km simulations at 25km resolution is actually departing from the resolution of the driving data.

As a final note, Supplementary Figs. 11 and 12 show the variations of the 1.5km and 2.2km CPM projections across different horizontal scales. In addition to the 12km and 25km, we have added projections at the CPM native (1.5/2.2km) and 60km scale[3] to the analysis. The 2.2km projected changes above the 99.9 percentile at the 60km scale are clearly positive but somewhat less than the projected increase at the 25km scale. For percentiles above 99.0, the projected future increase at the 2.2km native scale by the 2.2km CPM is actually smallest, but clearly positive. It is the opposite for the 1.5km model projections, in which the highest increases are found at the native 1.5km scale and go to zero as the horizontal scales increase. Overall, the projections for both models appear to show different asymptotic behaviour at the larger horizontal scales, but they also have their own different projection estimates at scales below driving GCM scales.

2 Understanding the uncertainty in the 12km simulations

Certain assumptions are made during bootstrapping and spatial pooling, and they can be used to understand the uncertainties for the 12km model projections. Firstly, pooling assumes events have no spatial correlation. Secondly, inter-annual variability and rarity of outlier events are hard to assess with low number of model years; bootstrapping is supposed to address the former issue ($\binom{2\times 10-1}{10} = 92378$ possible year combinations for 10-yr bootstrap), but it cannot address the problem with outliers.

The projected increase in extreme precipitation in the 12km model is traced back to two consecutive model JJA days with widespread extreme precipitation in the future-climate simulation. The daily 1hr maximum and daily total precipitation from those two 12-km

[3] 60km is the resolution of the driving GCM for the 1.5km simulation; hourly precipitation is not available from the 60km GCM.

future-climate model days are shown in Supplementary Fig. 13 – a wide area across the Southeast England with peak hourly intensities exceeding 50mm/hr and daily totals exceed 100mm/dy. To highlight the horizontal scale of the event, daily totals exceeding 100 mm from Kent northward to the East Midlands – a distance of ≈ 300km. Daily precipitation extremes of such large horizontal scale are not unprecedented for the UK but not with such hourly intensities [Prior and Beswick, 2008]. The physical realism of such an event is questionable: such large intensities imply grid point storms, and the cumulus parameterisation grey zone has collapsed [Molinari and Dudek, 1992].

The blue line in Supplementary Fig. 10 illustrate the effect of removing the above two model days (“`12_outlierX`”) by treating them as missing data. The 12km projected future exceedance change becomes negligible when the outlier is removed, showing the outliers are having a major impact on the 12km results. The uncertainties are also greatly reduced when the outliers are removed. In Kendon et al [2014], simulations with additional aerosol physics were used, and these simulations showed no future increase (yellow line, “`12A`”, Supplementary Fig. 10). Projections for the 1.5km simulations with additional aerosol physics (purple line, “`1p5A`”) are generally similar to the one without except for the 99.999 percentile. Replicating the methodology used in Kendon et al [2014] with the simulations without aerosol physics (Supplementary Fig. 14) show a future increase in 12km precipitation extremes, although this is not statistically significant. Hence the differences between here and Kendon et al [2014] are not caused by differences in analysis methodology, but the presence of an extreme outlying event in one 12km simulation but not in the other similar simulation with additional aerosol physics.

We count the number of 12km grid boxes with daily maximum hourly precipitation exceeding 25mm/hr for the 12km, 2.2km, and 1.5km present- and future-climate simulations (Supplementary Table 3); results from the 12km simulation with aerosol physics are included (i.e. the “(A)” simulations). Consistent with the projections, the two future-climate CPM simulations show higher counts than the present-climate simulation. All simulations show inter-annual variability for the counts; some JJAs have even zero counts. The 12km future-climate simulation show a future count increase, and stands out with the high count concentration in one single model JJA; the 87 counts in year 8 are all from the above two-day event, and accounts for ≈ 80% of all counts. Both present- and future-climate 12km simulations with aerosol physics also show high count concentrations in specific years, but the future projections show a total count decrease; this is in contrast with the total count increase for the non-aerosol 12km simulations. The only non-12km simulation with high single-year count concentration is the 1.5km present-climate simulation in which one JJA accounts for ≈ 50% of the counts. All other simulations (including the present-climate 12km simulation) have less single-season dominance. To highlight the higher robustness of the 2.2 and 1.5km model projections, the future count increase signals for both models are still preserved after the removal of the JJA with the highest number of counts. In contrast, if one removes the highest count years from the 12km simulation, this will introduce large changes to the projection.

As a final note, the probability of not drawing the outlier at all during bootstrap is $(1 - \frac{1}{10})^{10} \approx 35\%$ with 10 model years. This may look like a high probability; however, $\lim_{n\to\infty}(1 - \frac{1}{n})^n = e^{-1} \approx 36.8\%$. Hence, we do not know how (un)lucky we are in having the outlier; there are not enough model years to properly assess the rarity of the outlier.

References

Berthou S, Kendon EJ, Chan SC, Ban N, Leutwyler D, Schär C, Fosser G (2018) Pan-european climate at convection-permitting scale: a model intercomparison study. Clim Dyn (), DOI 10.1007/s00382-018-4114-6

Chan SC, Kendon EJ, Fowler HJ, Blenkinsop S, Roberts NM (2014a) Projected increases in summer and winter UK sub-daily precipitation extremes from high resolution regional climate models. Environ Res Lett 9():084,019, DOI 10.1088/1748-9326/9/8/084019

Chan SC, Kendon EJ, Fowler HJ, Blenkinsop S, Roberts NM, Ferro CAT (2014b) The value of high-resolution Met Office regional climate models in the simulation of multi-hourly precipitation extremes. J Climate 27(16):6155–6174, DOI 10.1175/JCLI-D-13-00723.1

Chan SC, Kahana R, Kendon EJ, Fowler HJ (2018) Projected changes in extreme precipitation over Scotland and northern England using a high-resolution regional climate model. Clim Dyn (), DOI 10.1007/s00382-018-4096-4

Dee DP, Uppala SM, Simmons AJ, Berrisford P, Poli P, Kobayashi S, Andrae U, Balmaseda MA, Balsamo G, Bauer P, Bechtold P, Beljaars ACM, van de Berg L, Bidlot J, Bormann N, Delsol C, Dragani R, Fuentes M, Geer AJ, Haimberger L, Healy SB, Hersbach H, Hölm EV, Isaksen L, Kallberg P, Köhler M, Matricardi M, McNally AP, Monge-Sanz BM, Morcrette JJ, Park PK, Peubey C, de Rosnay P, Tavolato C, Thêpaut JN, Vitart F (2011) The ERA-Interim reanalysis: configuration and performance of the data assimilation system. Q J R Meteorol Soc 137(656):553–597, DOI 10.1002/qj.828

Donlon C, Martin M, Stark J, Roberts-Jones J, Fiedler E, Wimmer W (2012) The operational sea surface temperature and sea ice analysis (OSTIA) system. Remote Sensing Environ 116():140–158, DOI 10.1016/j.rse.2010.10.017

Fosser G, Kendon EJ, Chan SC, Lock A, Roberts N (submitted) Optimal configuration and resolution for the first convection permitting ensemble of climate projections over the uk? Int J Climatol ()

Hanley KE, Plant RS, Stein THM, Hogan RJ, Nicol JC, Lean HW, Halliwell C, Clark PA (2015) Mixing length controls on high resolution simulations of convective storms. Q J R Meteorol Soc 141(686):272–284, DOI 10.1002/qj.2356

Harrison DL, Driscoll SJ, Kitchen M (2000) Improving precipitation estimates from weather radar using quality control and correction techniques. Meteorol Appl 7:135–144, DOI 10.1017/S1350482700001468

Kendon EJ, Jones RG, Kjellström E, Murphy JM (2010) Using and designing GCM-RCM ensemble regional climate projections. J Climate 23:6485–6503, DOI 10.1175/2010JCLI3502.1

Kendon EJ, Roberts NM, Senior CA, Roberts MJ (2012) Realism of rainfall in a very high resolution regional climate model. J Climate 25:5791–5806, DOI 10.1175/JCLI-D-11-00562.1

Kendon EJ, Roberts NM, Fowler HJ, Roberts MJ, Chan SC, Senior CA (2014) Heavier summer downpours with climate change revealed by weather forecast resolution model. Nature Climate Change 4:570–576, DOI 10.1038/nclimate2258

Lewis E, Quinn N, Blenkinsop S, Fowler HJ, Freer J, Tanguy M, Hitt O, Coxon G, Bates P, Woods R (2018) A rule based quality control method for hourly rainfall data and a 1km resolution gridded hourly rainfall dataset for great britain: Ceh-gear1hr. J Hydrol 564():930–943, DOI 10.1016/j.jhydrol.2018.07.034

Mizielinski MS, Roberts MJ, Vidale PL, Schiemann R, Demory ME, Strachan J, Edwards T, Stephens A, Lawrence BN, Pritchard M, Chiu P, Iwi A, Churchill J, Novales CDC, Kettleborough J, Roseblade W, Selwood P, Foster M, Glover M, Malcolm A (2014) High

resolution global climate modelling; the UPSCALE project, a large simulation campaign. Geosci Model Devel 7():1629–1640, DOI 10.5194/gmd-7-1629-2014

Molinari J, Dudek M (1992) Parameterization of convective precipitation in mesoscale numerical models: A critical review. Mon Weather Rev 120(2):326–344, DOI 10.1175/1520-0493(1992)120⟨0326:POCPIM⟩2.0.CO;2

Perry M, Hollis D, Elms M (2009) The Generation of Daily Gridded Datasets of Temperature and Rainfall for the UK. Met Office National Climate Information Centre, FitzRoy Road, Exeter, Devon EX1 3PB, United Kingdom

Prior J, Beswick M (2008) The exceptional rainfall of 20 july 2007. Weather 63(9):261–267, DOI 10.1002/wea.322

Williamson DL (2013) The effect of time steps and time-scales on parametrization suites. Q J R Meteorol Soc 139(671):548–560, DOI 10.1002/qj.1992

Supplementary Table 1 List of UKMO 1.5km CPM, 12km Euro-CORDEX and 60km "N216" GCM simulations used in the original UK-only simulations. Their UM versions, simulation domains, horizontal size of grid boxes (H-Res) are also given. The last column either gives the lateral boundary conditions (LBC) for the CPM simulations or the driving SSTs for GCM simulations. The same model years (present: 1999–2008; future: 2099–2108) are analysed. Similar to the information shown in Table 1 in the main text, all hindcast simulations use the Gregorian calendar and all present- and future-climate simulations use the 360-day calendar.

Simulation	UM ver.	Domain	H-Res (km)	LBC (RCM) / SST (GCM)
60km present	7.7	Global	60	Daily OSTIA SST [Donlon et al, 2012]
60km future	7.7	Global	60	Daily OSTIA SST + change[1]
1.5km present-N [Chan et al, 2018]	7.8	NUK	1.5	60km HadGEM3 present [Mizielinski et al, 2014][2]
1.5km future-N [Chan et al, 2018]	7.8	NUK	1.5	60km HadGEM3 future[2]
1.5km hindcast-S [Kendon et al, 2012]	7.6	SUK	1.5	ERA-Interim [Dee et al, 2011][2]
1.5km present-S [Kendon et al, 2014]	7.8	SUK	1.5	60km HadGEM3 present[2]
1.5km future-S [Kendon et al, 2014]	7.8	SUK	1.5	60km HadGEM3 future [Mizielinski et al, 2014][2]
12km hindcast	7.6	Europe	12	ERA-Interim[3]
12km present	7.8	Europe	12	60km HadGEM3 present[3]
12km future	7.8	Europe	12	60km HadGEM3 future[3]

Supplementary Table 2 "Secondary simulations" that are used for specific analyses [Fosser et al, submitted].

Simulation	UM ver.	Domain	H-Res (km)	LBC
"Small-domain" 2.2km hindcast	10.1	UK	2.2	ERA-Interim [Dee et al, 2011][2]
"New" 1.5km hindcast	10.1	UK	1.5	ERA-Interim [Dee et al, 2011][2]

Supplementary Table 3 The number of SUK JJA grid points with daily maximum hourly precipitation exceeding 25 mm/h for each model year of the climate change simulations. "P" and "F" indicate present- and future-climate simulation respectively. "(A)" indicate the 12km simulations with additional aerosol physics. Total counts and percentage contributed by the large year are shown at the bottom of the table.

	12P	12F	12P(A)	12F(A)	2.2P	2.2F	1.5P	1.5F	1.5P(A)	1.5F(A)
Y0 "1998"	5	0	0	0	0	15	0	10	1	12
Y1 "1999"	0	2	0	0	0	22	12	11	11	9
Y2 "2000"	9	14	7	42	2	30	1	1	4	0
Y3 "2001"	8	0	84	1	7	27	7	10	24	15
Y4 "2002"	12	0	2	0	1	0	15	10	10	12
Y5 "2003"	0	0	2	0	9	119	0	19	0	25
Y6 "2004"	6	0	21	0	6	11	4	3	0	9
Y7 "2005"	0	3	0	0	3	0	0	30	0	11
Y8 "2006"	0	87	0	0	7	65	42	54	3	35
Y9 "2007"	0	0	0	0	11	0	0	18	0	3
Total	40	106	116	43	46	289	81	166	53	131
$\%_{\mathrm{LargestYr}}$	30	82	72	98	20	41	52	33	45	27

[1] RCP8.5 greenhouse gas for 2099–2108, SST 1999–2008 with projected change for the end of 21st century superposed [Mizielinski et al, 2014].

[2] An intermediate 12km EuroCORDEX simulation is used.

[3] This is the intermediate 12km EuroCORDEX simulation for 1.5km CPM simulation.

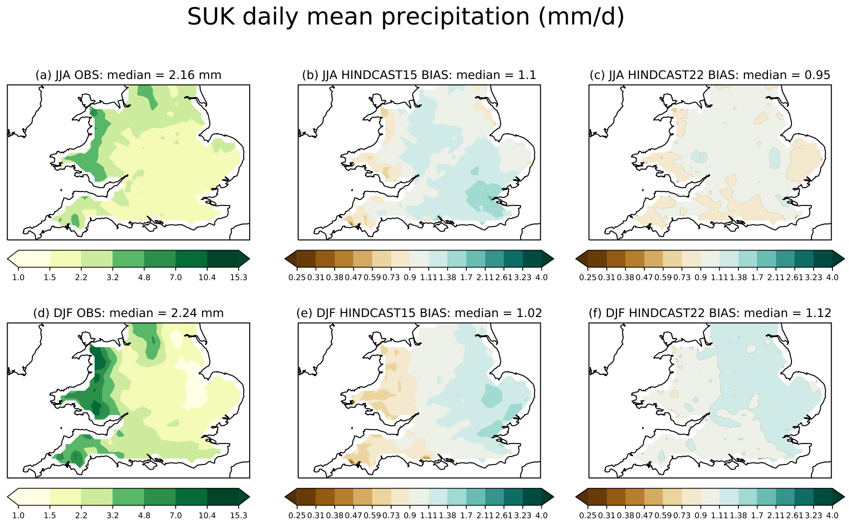

Supplementary Fig. 1 Mean biases of the 1.5km (centre column) and 2.2km (right column) SUK hindcasts for JJA (upper row) and DJF (bottom row). UK gridded daily observations [left column; Perry et al, 2009] are used to diagnose the mean biases. All presented results (including observations) are for 1999–2008 (i.e. the 2.2km hindcast years).

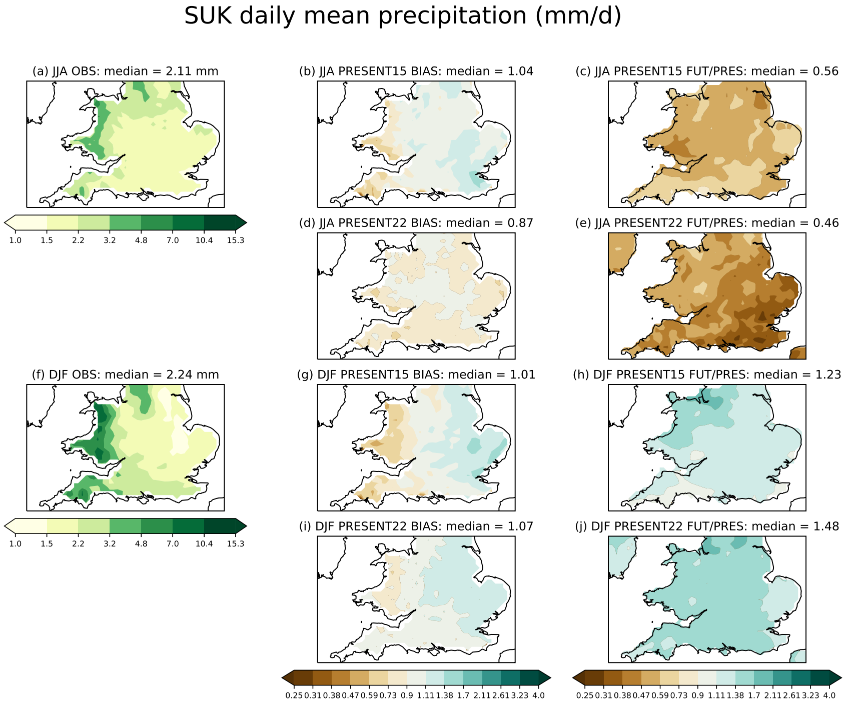

Supplementary Fig. 2 Biases and projected change for SUK JJA (panels b to e) and DJF (panels g to j) mean daily precipitation for the 1.5km and 2.2km CPM. The biases are compared against UK gridded daily observations [panels a and f; Perry et al, 2009]. The observation baseline period is 1998 to 2007 (same as in the underlying driving SST period).

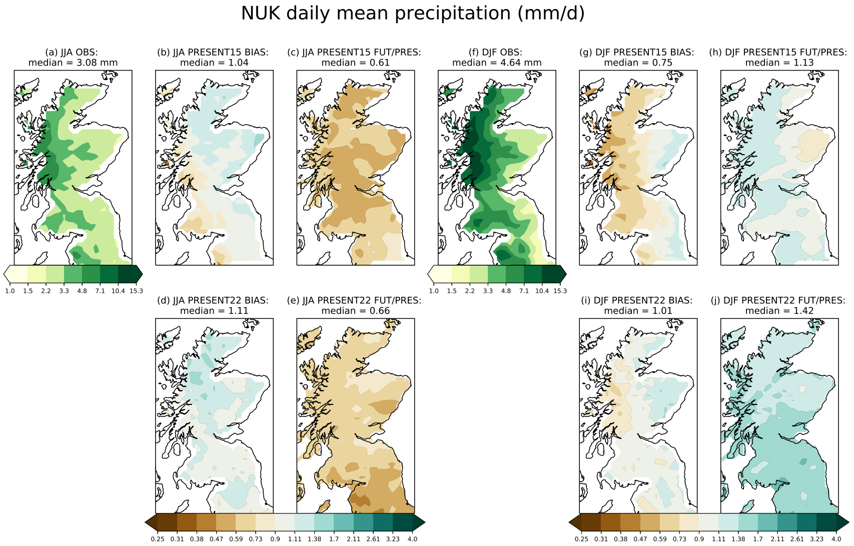

Supplementary Fig. 3 Same as in Fig. 2, but for Northern UK (NUK) instead.

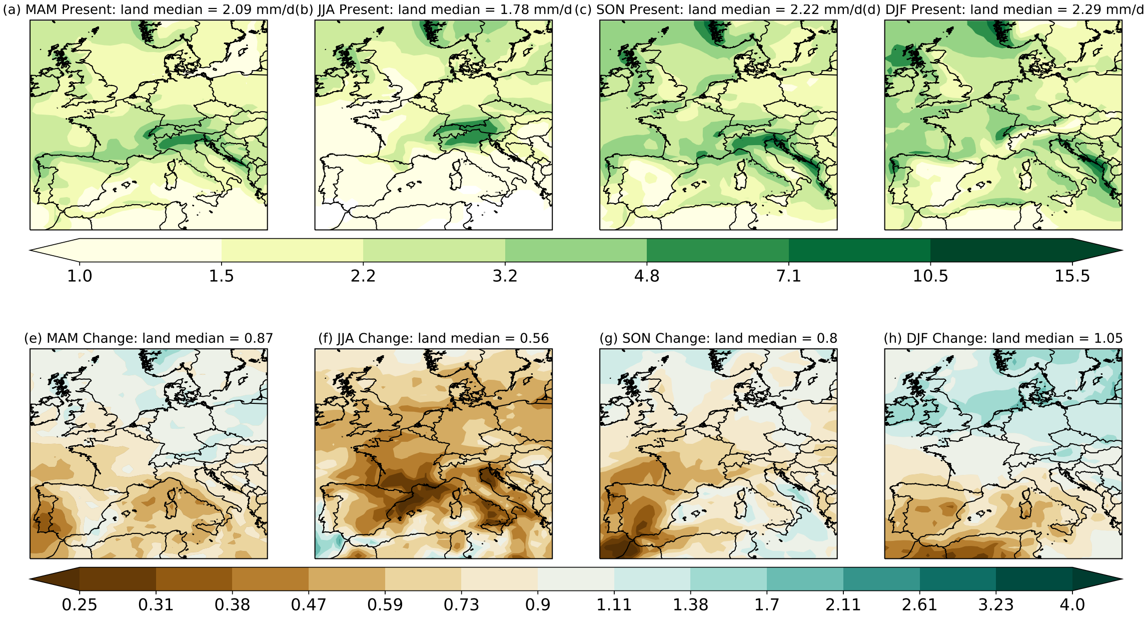

Supplementary Fig. 4 Present-climate seasonal means and future projected change for the 60km "N216" GCM simulations. The panels are (a,e) March-April-May (MAM), (b,f) June-July-August (JJA), (c,g) September-October-November, and (d,h) December-January-February (DJF).

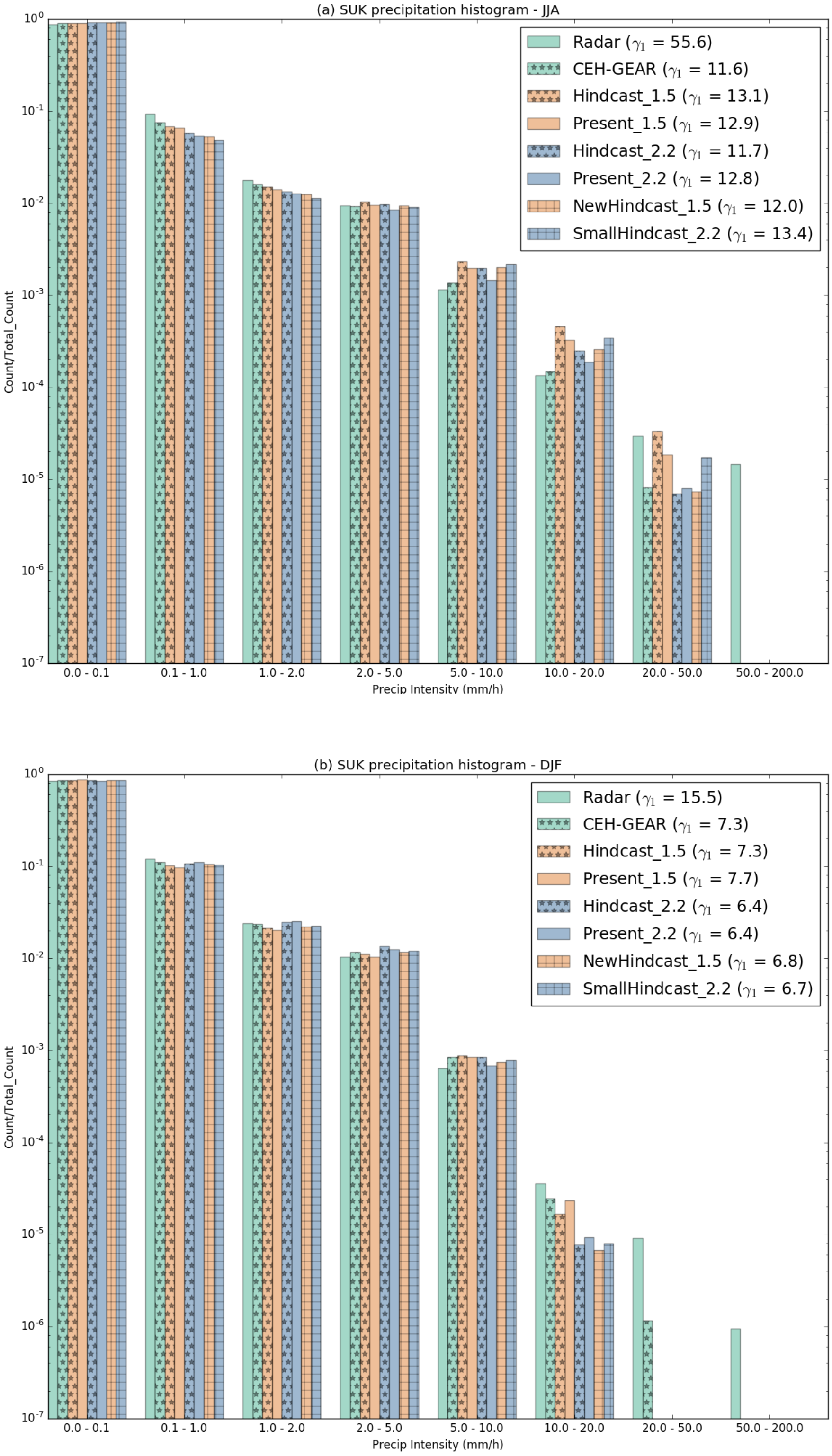

Supplementary Fig. 5 Following Chan et al [2018], the histogram of (a) JJA and (b) DJF hourly precipitation intensities over SUK for the 1.5/2.2km CPM hindcast/present-climate simulations and observations (radar and CEH-GEAR1hr). The skewness of the precipitation intensities are given in the legend. All data are regridded to a common 12km grid.

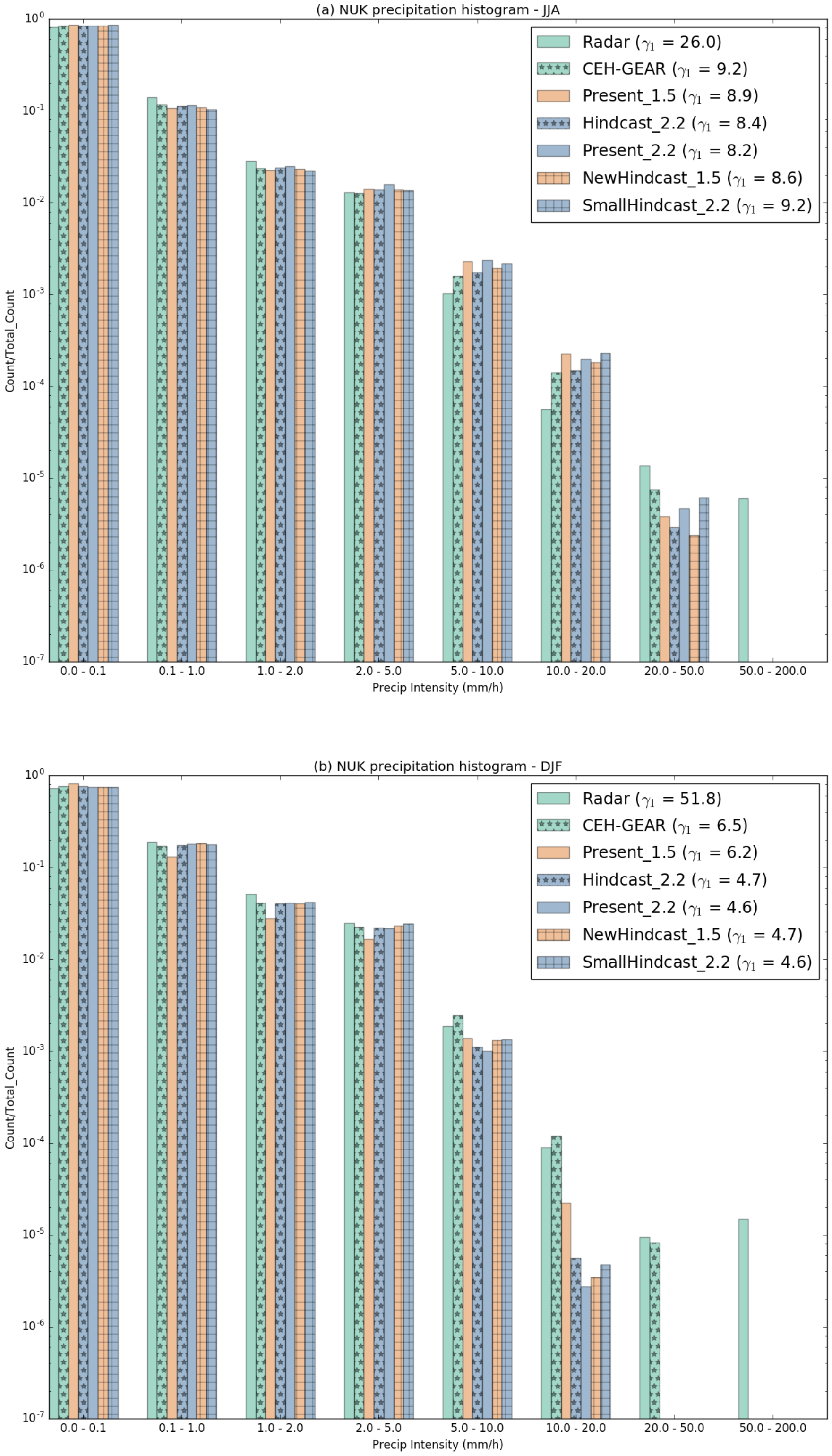

Supplementary Fig. 6 Same as in Main Fig. 5, but for Northern UK. Note, 1.5km hindcast results are not available for the Northern UK.

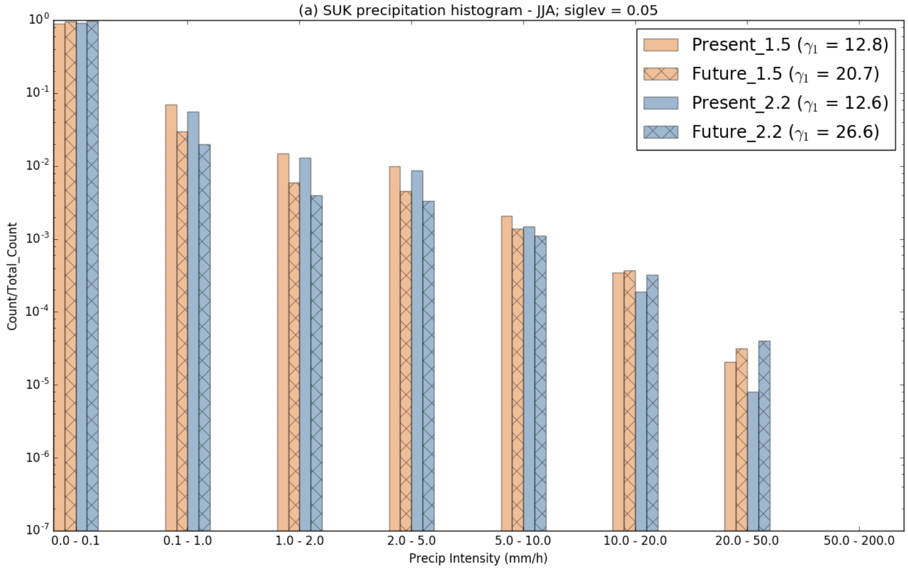

	0.0 - 0.1	0.1 - 1.0	1.0 - 2.0	2.0 - 5.0	5.0 - 10.0	10.0 - 20.0	20.0 - 50.0	50.0 - 200.0
1.5 fut/present	1.060*	0.430*	0.399*	0.458*	0.681*	1.073*	1.535*	inf
2.2 fut/present	1.054*	0.363*	0.304*	0.383*	0.737*	1.687*	5.043*	inf

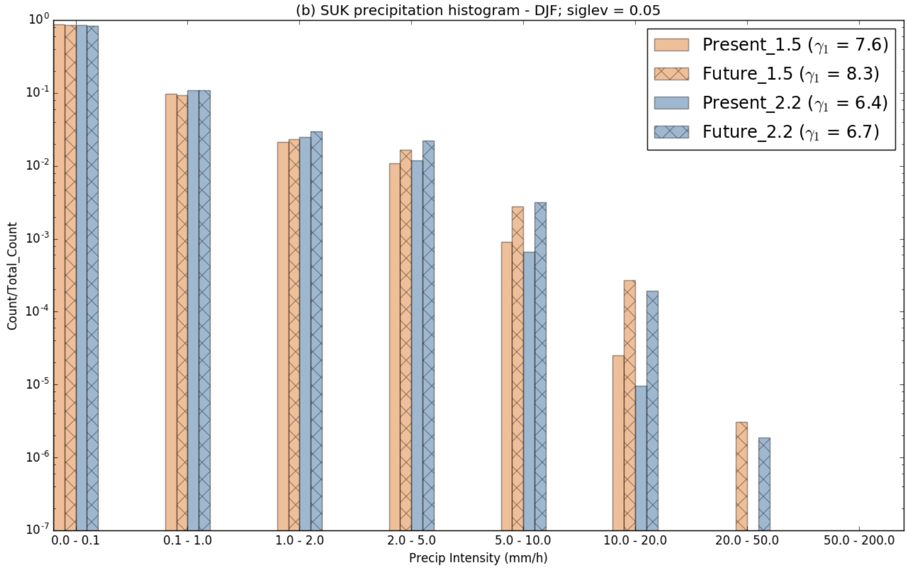

	0.0 - 0.1	0.1 - 1.0	1.0 - 2.0	2.0 - 5.0	5.0 - 10.0	10.0 - 20.0	20.0 - 50.0	50.0 - 200.0
1.5 fut/present	0.994*	0.953*	1.112*	1.532*	3.085*	10.661*	inf*	inf
2.2 fut/present	0.981*	0.983*	1.210*	1.854*	4.851*	19.794*	inf*	inf

Supplementary Fig. 7 Same as in Fig. 5, but SUK future change experiments. Future change ratios are shown in table below; "inf" indicates no counts in the present-climate simulation (i.e. "dividing-by-zero"). Changes that are significant at the 5% level are marked with asterisks; a two-tailed χ-squared tests are used with the exception when counts are below 10 in which Fisher's exact tests are used instead. Note that the significance tests are for the difference between the two simulations (i.e. Future minus Present) and not for the ratio between the two; hence, tests can be applied to the situation when the present-climate simulation has 0 counts.

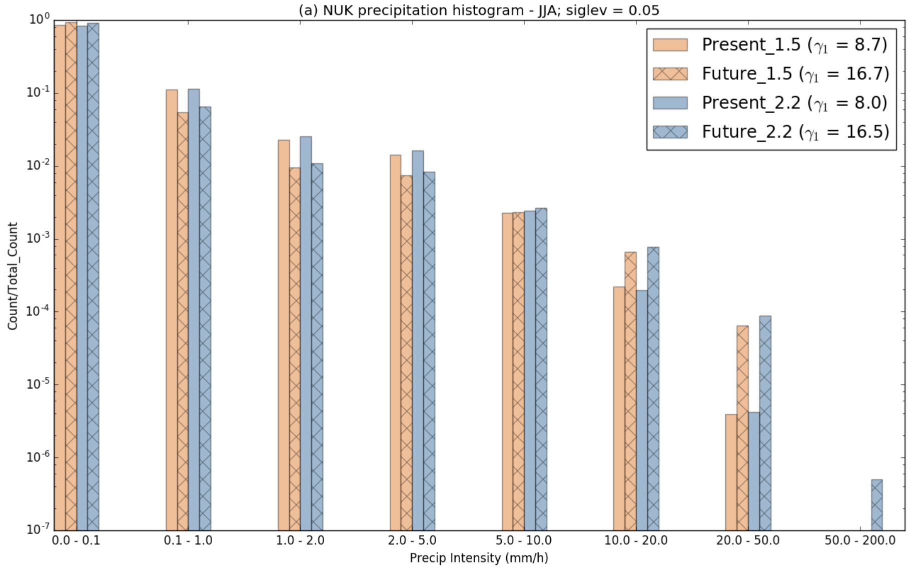

	0.0 - 0.1	0.1 - 1.0	1.0 - 2.0	2.0 - 5.0	5.0 - 10.0	10.0 - 20.0	20.0 - 50.0	50.0 - 200.0
1.5 fut/present	1.091*	0.489*	0.414*	0.523*	1.036*	3.021*	16.446*	inf
2.2 fut/present	1.085*	0.562*	0.428*	0.520*	1.097*	3.866*	21.179*	inf*

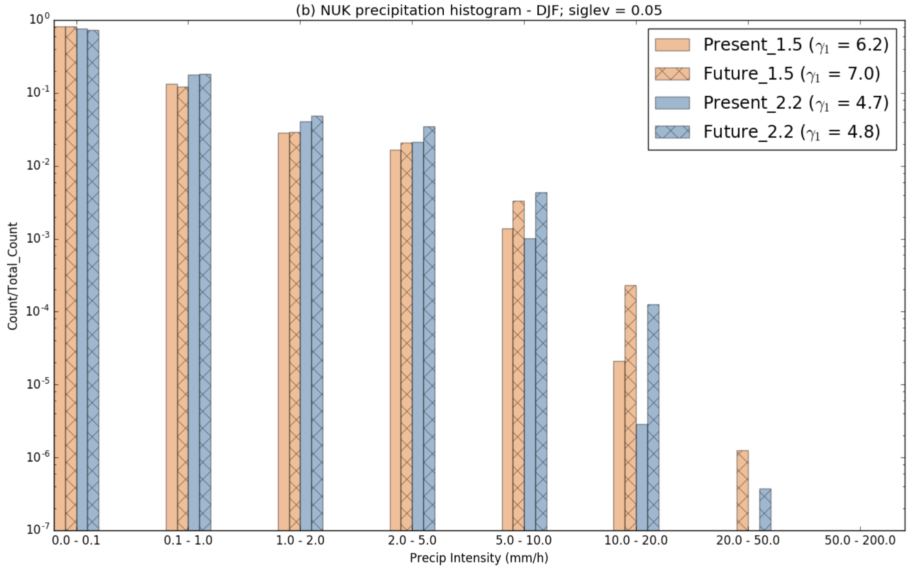

	0.0 - 0.1	0.1 - 1.0	1.0 - 2.0	2.0 - 5.0	5.0 - 10.0	10.0 - 20.0	20.0 - 50.0	50.0 - 200.0
1.5 fut/present	1.007*	0.909*	1.011*	1.253*	2.416*	10.950*	18.000*	inf
2.2 fut/present	0.965*	1.008*	1.197*	1.630*	4.302*	44.217*	6.000	inf

Supplementary Fig. 8 Same as in Main Fig. 7, but for Northern UK.

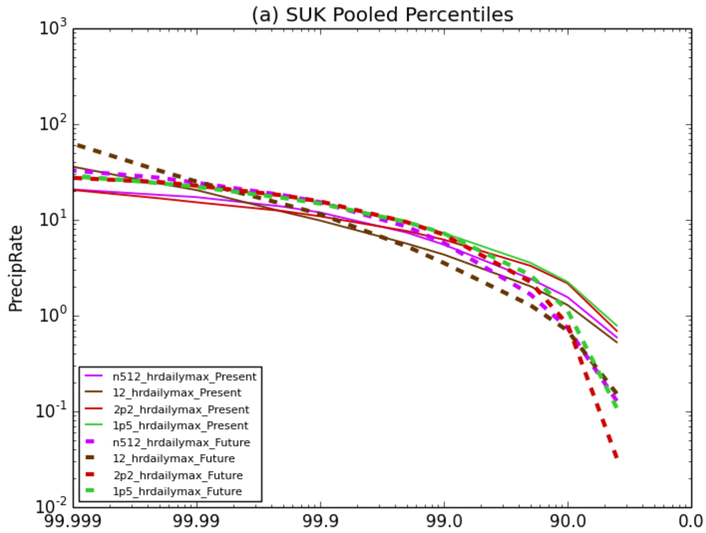

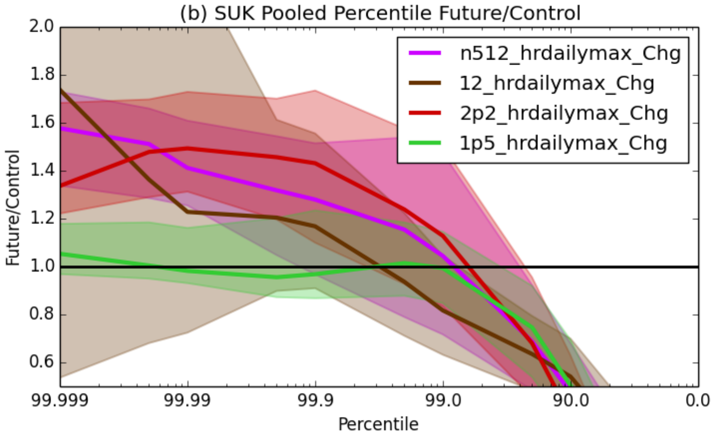

	99.999	99.995	99.99	99.95	99.9	99.5	99.0	95.0	90.0	75.0
n512	1.58*	1.51*	1.41*	1.32*	1.28*	1.15	1.04	0.69*	0.48*	0.22*
12	1.74	1.36	1.23	1.20	1.17	0.94	0.82	0.63*	0.54*	0.29*
2p2	1.34*	1.48*	1.49*	1.46*+	1.43*+	1.24^	1.13	0.68^	0.37*+	0.05*+
1p5	1.05+	1.00+	0.98+	0.96+	0.97	1.01	0.99	0.75^	0.50*	0.14*

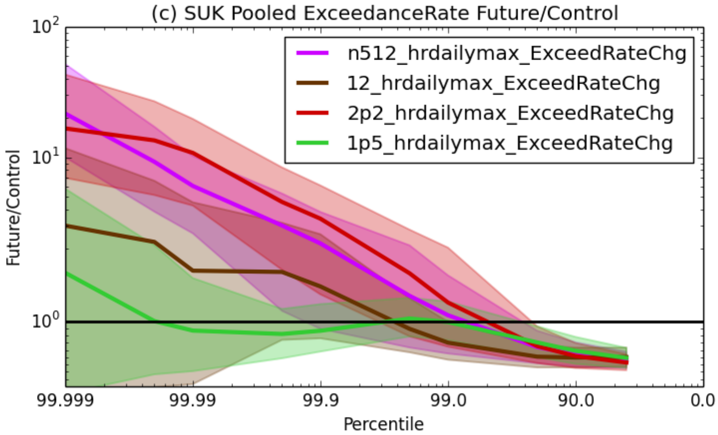

	99.999	99.995	99.99	99.95	99.9	99.5	99.0	95.0	90.0	75.0
n512	21.67*	9.25*	6.04*	3.01*	2.19*	1.35	1.08	0.64*	0.54*	0.46*
12	3.00*	2.25	1.70	1.68	1.48	0.89	0.70^	0.51*	0.50*	0.50*
2p2	16.67*	13.50*	10.83*	4.56*	3.39*	1.66^	1.26	0.65^	0.52*	0.43*
1p5	1.67	1.00	0.87	0.82	0.87	1.04	0.99	0.71*	0.59*	0.49*

Supplementary Fig. 9 All hour percentiles for data regridded to 25km (top panel) for the 25km "N512", 2.2km ("2p2"), 1.5km ("1p5") and 12km present- and future climate simulations. Middle panel shows the change of the same quantile in the future simulation with the year-block-boostrap-estimated 95% confidence interval shaded; the actual change ratio is given the table below. Future changes not rejected at the 5% and 10% level from the present-climate baseline are marked with "*" (asterisk/star) and "^" (caret) respectively. A "+" (plus) sign is used to mark changes that are significantly different from the 25km N512 GCM future change at the 5% level. Bottom panel is for changes in present-climate exceedance rates in the future simulation. Data are regridded to a common 25km "N512" grid before analysis.

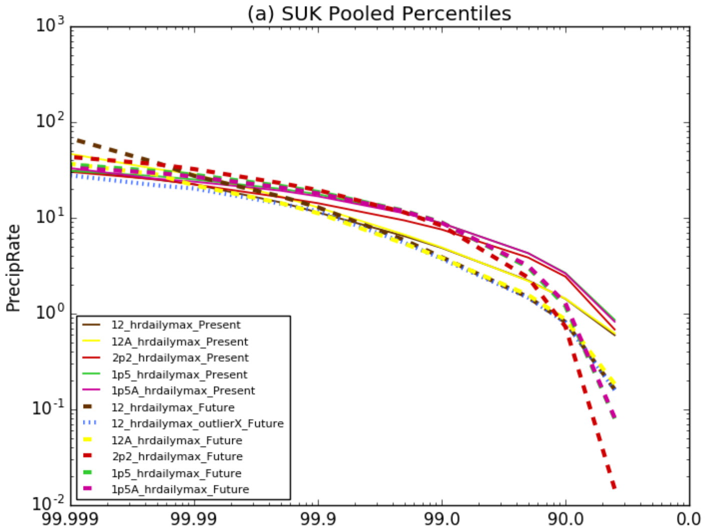

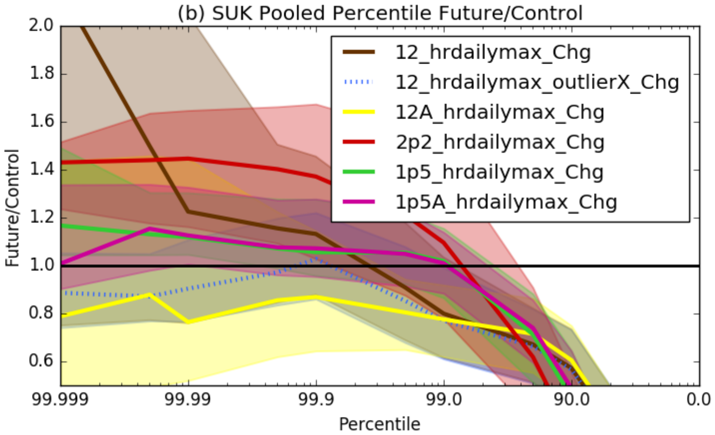

	99.999	99.995	99.99	99.95	99.9	99.5	99.0	95.0	90.0	75.0
12	2.16*	1.50	1.23	1.16	1.13	0.91	0.80	0.67*	0.57*	0.27*
12_outlierX	0.89	0.87^	0.90	0.97	1.03	0.85	0.77*	0.66*	0.57*	0.27*
12A	0.79	0.88	0.76	0.86	0.87	0.80*	0.78*	0.72*	0.61*	0.30*
2p2	1.43*	1.44*	1.45*	1.40*	1.37*	1.21	1.10	0.62^	0.30*	0.02*
1p5	1.17	1.13^	1.12^	1.07	1.06	1.05	1.02	0.71*	0.45*	0.09*
1p5A	1.01	1.15^	1.13	1.08	1.07	1.05	1.01	0.74*	0.49*	0.10*

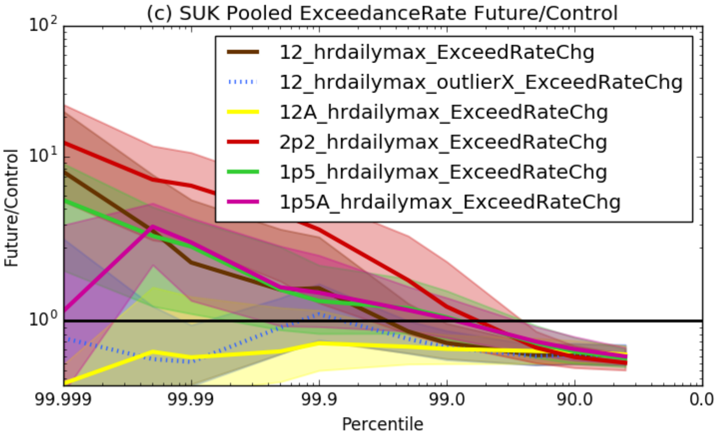

	99.999	99.995	99.99	99.95	99.9	99.5	99.0	95.0	90.0	75.0
12	7.62*	2.69^	1.79	1.42	1.44	0.84	0.68^	0.52*	0.53*	0.51*
12_outlierX	0.75	0.46	0.42	0.90	1.08	0.74^	0.63*	0.50*	0.52*	0.51*
12A	0.12	0.56	0.49	0.57	0.68	0.63^	0.60*	0.57*	0.57*	0.53*
2p2	12.78*	6.64*	5.96*	3.63*	2.75*	1.55^	1.19	0.62*	0.49*	0.41*
1p5	4.62*	2.46*	2.04*	1.42^	1.26	1.16	1.04	0.69*	0.57*	0.48*
1p5A	1.12	2.90*	2.19*	1.45*	1.38*	1.14	1.02	0.70*	0.60*	0.50*

Supplementary Fig. 10 Same as in Main Fig. 9, but with data regridded to 12km grid and 25km "N512" results excluded (hence no "+" marks). For the 12km model projections, additional results from outlier removal; blue short dashes, "outlierX") and aerosol physics ("12A", yellow line; "1p5A", purple line) are included. Note that even the 95% CI for 2.2km projections for the bottom panel are well above the no-change "1" line, the actual p values are not significant at the 5% level due to positive skew for the CI due to the way we estimate the null distribution to be (see Section 3.1). The non-rejection at 5% level is often borderline; for instance, the p-value for 99.999 is 0.031, which is barely above the 0.025 two-tail requirement.

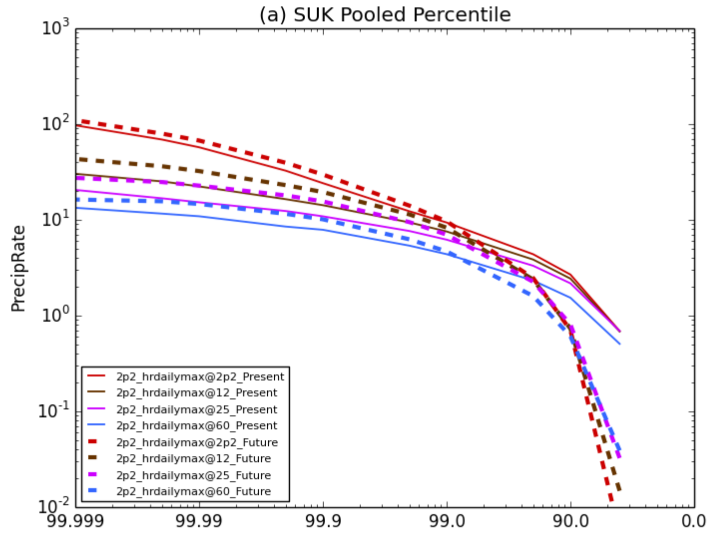

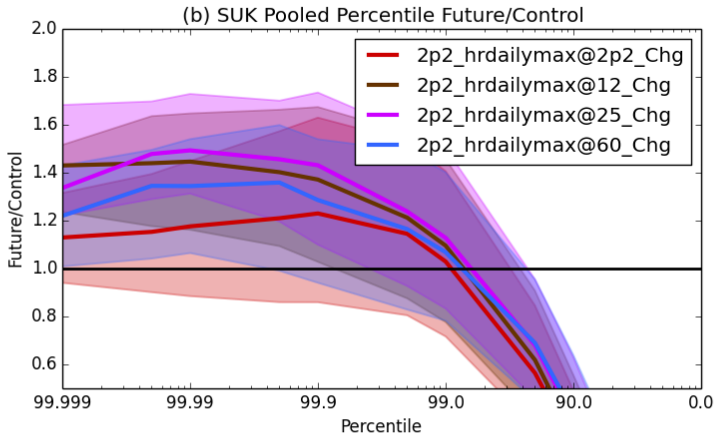

	99.999	99.995	99.99	99.95	99.9	99.5	99.0	95.0	90.0	75.0
2p2	1.13	1.15	1.18	1.21	1.23	1.15	1.03	0.56*	0.26*	0.01*
12	1.43*	1.44*	1.45*	1.40*	1.37*	1.21	1.10	0.62^	0.30*	0.02*
25	1.34*	1.48*	1.49*	1.46*	1.43*	1.24^	1.13	0.68^	0.37*	0.05*
60	1.22*	1.35*	1.34*	1.36*	1.29*	1.16	1.07	0.69^	0.40*	0.08*

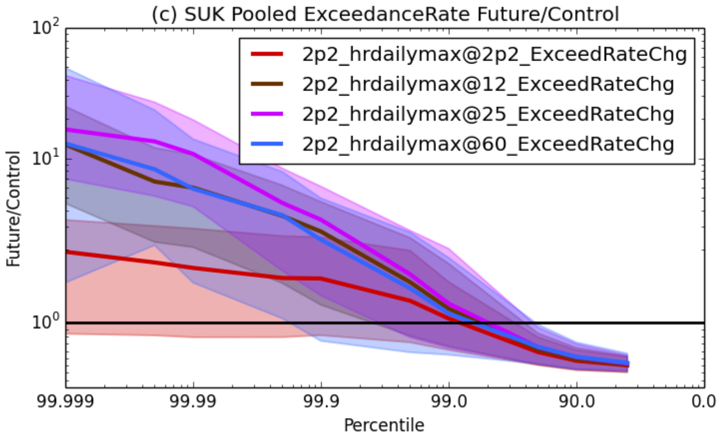

	99.999	99.995	99.99	99.95	99.9	99.5	99.0	95.0	90.0	75.0
2p2	1.97*	1.82*	1.75*	1.61*	1.60*	1.29	1.05	0.59*	0.47*	0.40*
12	12.78*	6.64*	5.96*	3.63*	2.75*	1.55^	1.19	0.62*	0.49*	0.41*
25	16.67*	13.50*	10.83*	4.56*	3.39*	1.66^	1.26	0.65^	0.52*	0.43*
60	13.00*	8.25*	5.86*	3.67*	2.38*	1.47	1.12	0.66^	0.52*	0.44*

Supplementary Fig. 11 Same as in Supplementary Fig. 10, but for 2.2km CPM SUK projections only across different horizontal scales – from native 2.2km to regridded 12km, 25km and 60km.

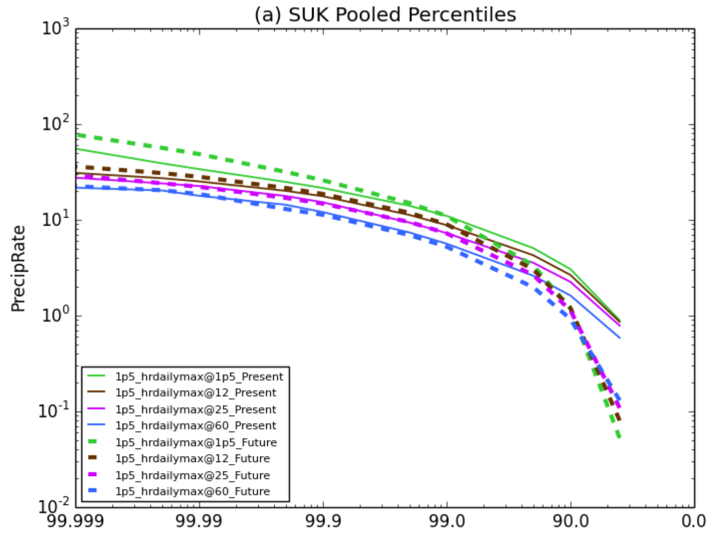

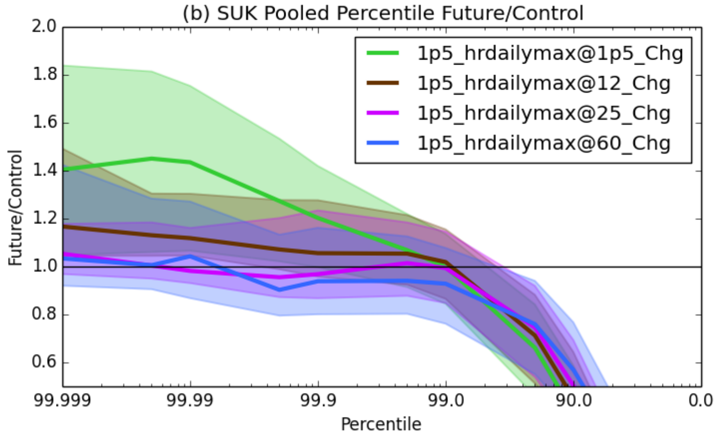

	99.999	99.995	99.99	99.95	99.9	99.5	99.0	95.0	90.0	75.0
1p5	1.40*	1.45*	1.44*	1.27*	1.20*	1.07	1.00	0.66*	0.39*	0.06*
12	1.17	1.13^	1.12^	1.07	1.06	1.05	1.02	0.71*	0.45*	0.09*
25	1.05	1.00	0.98	0.96	0.97	1.01	0.99	0.75^	0.50*	0.14*
60	1.03	1.01	1.04	0.90	0.94	0.94	0.93	0.76	0.57*	0.22*

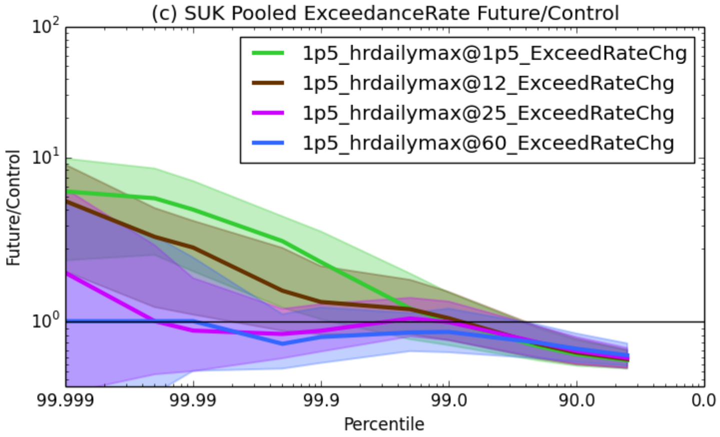

	99.999	99.995	99.99	99.95	99.9	99.5	99.0	95.0	90.0	75.0
1p5	5.49*	4.86*	3.98*	2.28*	1.81*	1.17	1.00	0.65*	0.54*	0.46*
12	4.62*	2.46*	2.04*	1.42^	1.26	1.16	1.04	0.69*	0.57*	0.48*
25	1.67	1.00	0.87	0.82	0.87	1.04	0.99	0.71*	0.59*	0.49*
60	1.00	1.00	1.00	0.69	0.78	0.84	0.85	0.70^	0.62*	0.53*

Supplementary Fig. 12 Same as in Supplementary Fig. 11, but for the 1.5km CPM simulations only.

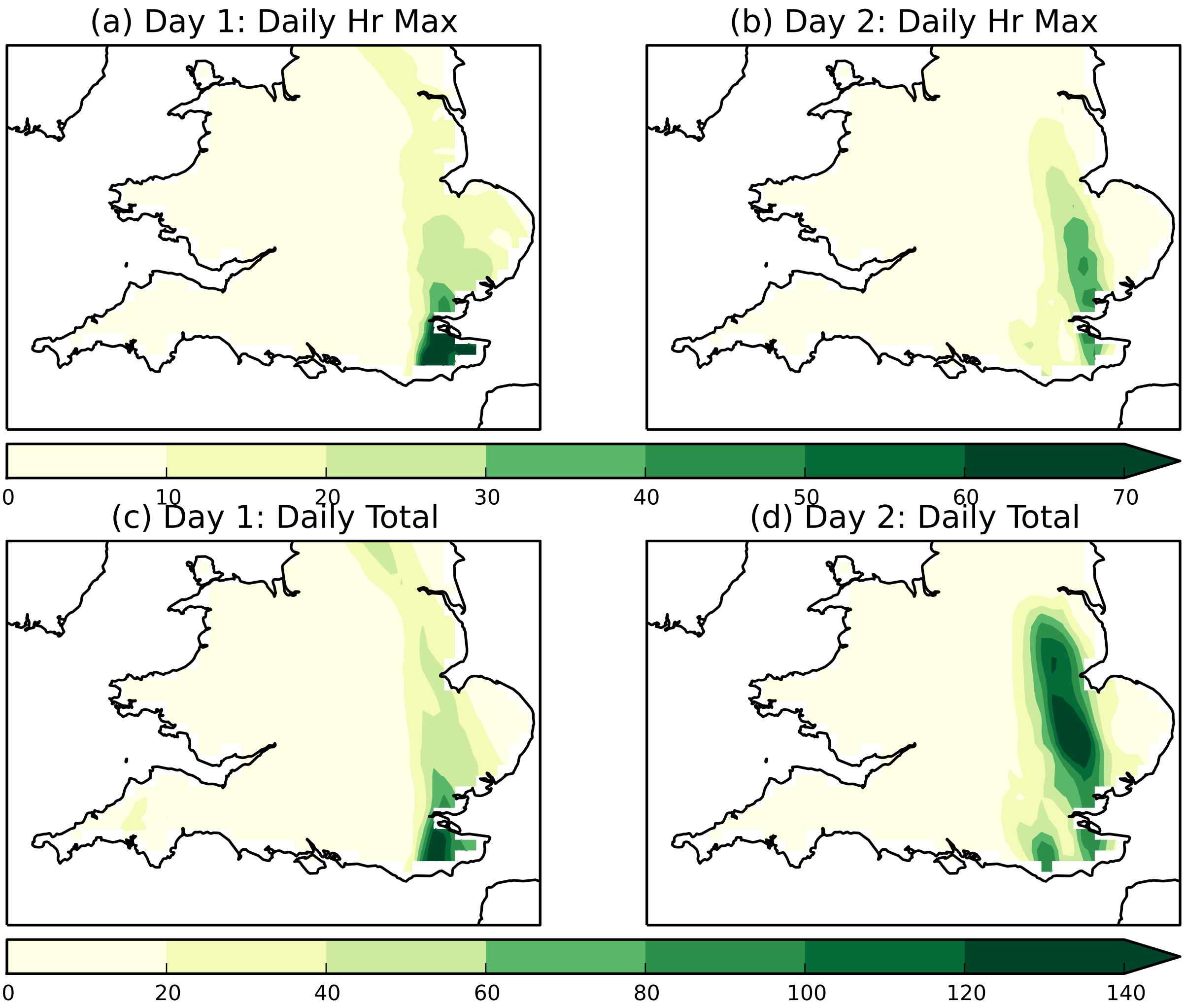

Supplementary Fig. 13 Daily maximum hourly (top panels) and total (bottom panels) precipitation for the very large two-day extreme event in the 12km future-climate simulation.

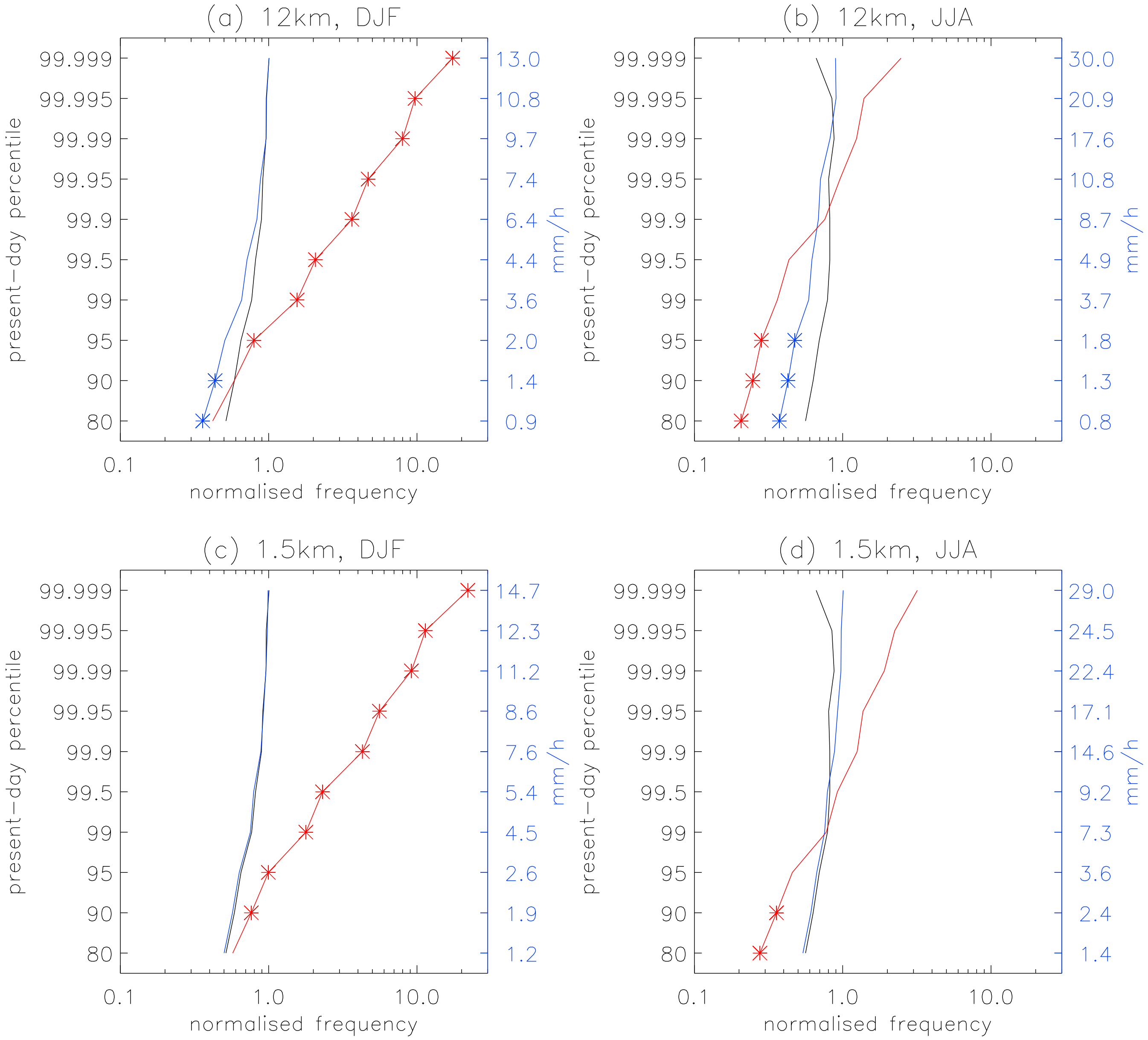

Supplementary Fig. 14 Same as in Fig. 5 in Kendon et al [2014], but with the 12km and 1.5km simulations without aerosol physics. Statistically significant changes are marked with asterisks "*".
